# CMV Status Drives Distinct Trajectories of CD4+ T Cell Differentiation

**DOI:** 10.3389/fimmu.2021.620386

**Published:** 2021-04-15

**Authors:** Weiwen Zhang, Anna B. Morris, Erica V. Peek, Geeta Karadkhele, Jennifer M. Robertson, Haydn T. Kissick, Christian P. Larsen

**Affiliations:** ^1^ Xiangya School of Medicine, Central South University, Changsha, China; ^2^ Emory Transplant Center, Department of Surgery, Emory University School of Medicine, Atlanta, GA, United States; ^3^ Department of Urology, Emory University School of Medicine, Atlanta, GA, United States

**Keywords:** CMV, CD4+ T cells, memory, differentiation, cytotoxicity, CD57, CD28

## Abstract

Cytomegalovirus (CMV) is one of the most commonly recognized opportunistic pathogens and remains the most influential known parameter in shaping an individual’s immune system. As such, T cells induced by CMV infection could have a long-term impact on subsequent immune responses. Accumulating evidence indicates that memory T cells developed during past bacterial and viral infection can cross-react with unrelated pathogens, including transplant antigens, and can alter responses to *de novo* infections, vaccines, cancers, or rejection. Therefore, careful examination of T cell responses elicited by CMV is warranted to understand their potentially beneficial or harmful roles in future major immune events. Our detailed exploration of the distribution, phenotype, TCR repertoire and transcriptome of CD4+ T cells within CMV seropositive healthy individuals using high-dimensional flow cytometry and single cell multi-omics sequencing reveals that CMV seropositivity has highly significant age-independent effects, leading to a reduction in CD4+ naïve T cells and an expansion of CD4+ effector memory T cells and CD45RA+ effector memory T cells. These induced CD4+ effector memory T cells undergo a specific differentiation trajectory resulting in a subpopulation of CD57+CD27-CD28-CD244+ CD4+ T cells with cytotoxic function and TCR oligoclonality for optimal controlled coexistence with cytomegalovirus. Through gene set enrichment analysis, we found that this subpopulation is similar to virus-specific CD8+ T cells and T cells that mediate acute rejection in patients using tacrolimus and belatacept, a selective costimulation blocker. Together, these data suggest that memory CD4+ T cells induced by cytomegalovirus are formed *via* a distinct differentiation program to acquire cytotoxic function and can be potentially detrimental to transplant patients adopting costimulation blockade immunosuppressive regimen.

## Introduction

CMV reactivation or primary infection is a major source of morbidity in individuals with HIV, primary and secondary immunodeficiencies, autoimmune disease, and recipients of organ and bone marrow transplantation. While the immune response to CMV has been extensively studied, our understanding of the long-term impact of CMV infection in healthy humans is incomplete. Brodin et al. identified CMV as one of the most influential sources of variation in the immune system of healthy humans (1). For example, CMV-specific T cells can occupy up to 20% of the CD8+ T cell memory pool in CMV seropositive healthy humans (2). Because of the broad impact of CMV on the human population, a careful assessment of induced memory T cell populations following infection is important in understanding the long-term impact of CMV.

Study of the memory CD8+ T cell compartment has defined this CMV-specific memory as distinct phenotypically and differentially (3). Specifically, CMV-specific CD8+ T cells have been referred to as senescent and replication-deficient, with loss of costimulatory molecules and gain of CD57 and CD244 (3). However, recent research has indicated that these cells maintain a specialized subset of functions (4, 5). While less extensively studied, CMV specific CD4+ memory T cells are also retained in large numbers and bear a similar “senescent” profile (6–8). In addition, these CD4+ T cells express cytotoxic molecules typical of CD8+ T cells and NK cells (6, 9, 10). In-depth characterization using single-cell omics of CMV-induced memory CD4+ T cell populations has yet to be done.

As memory T cells primed during prior bacterial and viral infections can cross-react with antigens from unrelated pathogens or transplanted tissue and influence the responses to *de novo* infections, vaccination, cancer, or rejection (11–13), study of CMV-specific memory T cells can illuminate potentially pathogenic or beneficial T cell populations. Interestingly, CMV has been shown to both impair influenza vaccine responses in the elderly and augment influenza vaccine responses in the young (14). Furthermore, transplantation models have shown that heterologously-primed memory from various bacteria and viruses is a source of resistance and failure of costimulation blockade approaches (11–13). Due to the ubiquitous nature of CMV in the human population, understanding CMV-induced memory T cell populations is likely to inform aspects of other disease states, such as whether these CMV-induced memory T cells alter responses to vaccines and transplantation.

In this study, we performed an in-depth analysis of the distribution, phenotype, and transcriptome of CD4+ T cells within CMV seropositive individuals using a multi-omics approach. We confirmed an enrichment of effector memory CD4+ T cells that express CD57 and CD244 but have little to no expression of CD27 or CD28. Via single cell transcriptomics, we reveal that this subset of CD4+ T cells in CMV seropositive individuals is enriched in genes associated with cytotoxicity, indicating a cytolytic, non-senescent, CMV-induced population of CD4+ T cells. Further assessment of this population in CMV seropositive individuals revealed limited TCR clonal diversity with a distinct differentiation pattern, therefore defining a subset of T cells that differentiated along a specific trajectory from a limited number of clones. Together, these data and our comparative gene set enrichment analysis highlight the similar phenotypes of CMV-associated CD4+ CTL and “risky” T cell phenotypes that have been implicated as a predictive biomarker of transplant rejection.

## Materials and Methods

### Ethics Statement

Healthy individuals and patients undergoing renal transplantation at Emory University Hospital were enrolled in an immune monitoring protocol approved of by the Institutional Review Board at Emory University (IRB00006248). Participants obtained written informed permission before incorporation in this research.

### Study Subjects

For flow cytometry data analysis, a total of 30 CMV-seropositive and 25 CMV-seronegative healthy humans were assessed (**Table 1**). CMV negative and positive subjects showed no significant differences in EBV status, age and sex. Blood samples were collected over the course of 16 months from 12/2017 to 3/2019. 4 pairs of age- and gender-matched CMV-seropositive and CMV-seronegative healthy humans from the flow cytometry cohort were selected for single cell RNA sequencing data analysis. Their blood was drawn and processed in 4 different batches. CMV-seropositivity is determined by a positive test for CMV IgG. The experimental and analytic workflow for cytometric and single-cell transcriptomics is depicted in **Supplemental Figure 1**. For the longitudinal analysis of CD4+CD57+ TEM/TEMRA subsets, we assessed 6 CMV-seropositive stable transplant recipients on belatacept and 5 CMV-seropositive healthy humans (**Table 2**). CMV+ transplant recipients and healthy individuals showed no significant differences in EBV status, age and sex. Blood samples from the transplant recipient cohort were collected at 2-month intervals within the time frame of 09/2018 to 03/2019. Blood samples from the healthy individual cohort were collected at 6-month intervals within the time frame of 09/2018 to 02/2020.

**Table 1:** Clinical characteristics of CMV+ and CMV- healthy human samples used.

Characteristic	CMV-, N = 25	CMV+, N = 30	P-value
**EBV Status**			0.2
EBV+	23 (92%)	30 (100%)	
EBV-	2 (8%)	0 (0%)	
Age	39 ± 12	45 ± 12	0.089
**Sex**			>0.9
Female	18 (72%)	21 (70%)	
Male	7 (28%)	9 (30%)	

Mean ± SD is shown.

**Table 2:** Clinical characteristics of CMV+ healthy human samples and CMV+ belatacept patient samples from the longitudinal study cohort used.

Characteristic	Healthy humans, N = 5	Belatacept patients, N = 6	P-value
**EBV Status**			>0.9
EBV+	5 (100%)	6 (100%)	
EBV-	0 (0%)	0 (0%)	
Age	48 ± 10	41 ± 15	0.342
**Sex**			>0.9
Female	4 (80%)	5 (83%)	
Male	1 (20%)	1 (17%)	

Mean ± SD is shown.

### Sample Acquisition and Preparation for Flow Cytometry

Blood samples were collected in Cyto-Chex BCT tubes. Surface staining was performed on whole blood cells. After red blood cell lysis, cells were incubated with surface marker antibodies specific for CD3 [BUV737], CD4 [BUV395], CD8 [BV711], CD14/CD20/Live-Dead Aqua [BV510], CD57 [FITC], CD27 [PerCP-Cy5-5], CD28 [PE-Cy7], CD244 [PE-Dazzle], CD11a [Alexa Fluor 700], PD-1 [BV421], CD45RA [APC-H7], CCR7 [PE], CD45RB [BV650], HLA-DR [BV605], CD38 [BV786]. For intracellular cytokine stimulation, blood samples were collected in CPT tubes and peripheral blood mononuclear cells (PBMCs) were isolated. PBMCs were then incubated for 8 hours at 37°C, 5% CO2 in the presence of 1ug/mL CMV peptides from pp65 and IEL (pp65 PepMix and IEL PepMix from JPT Innovative Peptide Solutions). GolgiStop (BD) was added in the last 6 hours of incubation. Following stimulation, cells were fixed and permeabilized (BD Fix/Perm kit), and then assessed *via* flow cytometry for cytokine production using IL-2 [BV605], TNF [BV650], and IFN*γ* [BV785]. Flow cytometric acquisition was carried out using a BD LSRFortessa cytometer with BD FACSDiva software.

### Flow Cytometry Data Analysis

Manual sequential gating was performed using FlowJo version 10 to analyze populations and determine markers of interest. Expression levels of 9 markers (CD57, CD27, CD28, CD244, CD11a, PD-1, HLA-DR, CD38, CD45RB) within memory subsets of CD4+ T cells that are defined by CD45RA and CCR7 expression characteristics were compared between CMV+ and CMV- healthy subjects.

### Sample Acquisition and Preparation for Single Cell RNA Sequencing

Blood samples were collected in CPT tubes. PBMCs were isolated from whole blood instantly after sample collection by means of density gradient centrifugation. CD4+ T cells were extracted using MACS negative CD4+ T cell isolation kit and then stained with the following TotalSeq type C feature barcode antibodies (Biolegend): CD57, CD27, CD28, CD244, CD45RA, CCR7, CD45RO, PD-1, CD25 and CD39. RNA, ADT, and VDJ library preparation and sequencing were completed using 10X Genomics workflow.

### RNA, ADT, and VDJ Library Preparation and Sequencing

Cellular suspensions were loaded on a 10X Chromium instrument. Gene expression libraries and feature barcoding libraries were prepared using Chromium Single Cell 3′ V3 Reagent Kits with Cell Surface Protein Feature Barcoding Technology for Cell Surface Protein (10X Genomics). VDJ libraries were prepared using Chromium Single Cell V(D)J V1 Reagent Kits (10X Genomics). The sequencing of gene expression libraries was conducted as a custom configuration (26x8x91bp) on the Illumina HiSeq3000 with a depth of 50,000 reads per targeted cell. The sequencing of VDJ libraries was conducted as PE150 on the Illumina HiSeq3000 with a depth of 5,000 reads per targeted cell. The sequencing of 10X feature barcode libraries was conducted as a custom configuration (26x8x25bp) on the Illumina HiSeq3000 with a depth of 5,000 reads per targeted cell. Junk cells with low read depth were removed and the remaining libraries were sequenced to a depth of 60M to 190M reads per sample in total.

### Computational Analysis

#### Flow Cytometry

##### Preprocessing Workflow to Acquire CD4+ T Cells for Downstream Analysis

FCS files from all subjects were loaded into R as a FlowSet object using R flowCore package. The data was compensated using the spill matrix embedded within the FCS files. estimateLogicle transformation method was employed to normalize the raw intensities of non-linear markers used in the gating strategy (CD3, CD4, CD8 and CD14/CD20/LiveDead). CD4+ T cells were identified with a series of user-defined gates that were automatically drawn by R openCyto package for each subject (**Supplemental Figure 2**).

##### High-Dimensional Flow Cytometry Analysis for CD4+ T Cells

Equal number of CD4+ T cells (~5,000) per subject were sampled and merged into a single FlowSet object. Raw marker intensities were transformed using arcsinh with cofactor 150. Batch effect was removed using normalizeBatch() function from R Cydar package. Expression values for each marker were centered and outlier cells with abnormal marker expression values outside the normal ranges were excluded. MDS plot generated by plotMDS() function from R limma package was utilized to detect outlier subjects with atypical overall marker expression patterns. No significant outlier subjects were identified (**Supplemental Figure 3**).

Unsupervised cell clustering was performed using R FlowSOM package. In FlowSOM the number of clusters is user-defined. Initially 35 cluster number was imposed (15). To find the optimal cluster number, clusters were grouped based on their similarity using hierarchical clustering by R basic function hclust(), the result of which was visualized by a dendrogram (**Supplemental Figure 4**). Clusters bearing similar expression patterns were merged and re-annotated, and as a result 16 clusters were recognized. Finally, flow cytometry data were represented by tSNE plots using R Rtsne package. To validate the concordance between conventional bimodal gating analysis and unsupervised clustering technique, R package CytoRSuite which provides an interactive interface for flow cytometry data analysis in R was used to manually gate cells with phenotypes of significantly different clusters. These cells were later projected onto the tSNE space colored by red with the rest of the cells grayed out (**Supplemental Figure 5**).

#### Single Cell RNA Sequencing

##### Gene and ADT Data

Fastq files, gene and ADT (Antibody-Derived Tags) expression matrix were generated using python Cell Ranger package. The pre-processing workflow is illustrated in **Supplemental Figure 6**; The Seurat preprocessing pipeline was used to remove rare transcripts, low-quality reads, and dying cells (16, 17). Next, an exploratory clustering step followed by analysis of transcript and protein expression was performed to exclude non-CD4+ T cell contaminants. Balanced numbers of clean high-quality CD4+ T cells from each sample were merged for downstream analysis. Seurat’s reference-based integration pipeline was applied on the merged gene dataset to achieve the following goals: 1) each individual dataset was normalized using SCTranform method; 2) 3000 genes exhibiting the highest cell-to-cell variation across all individual datasets were selected and set as anchors; 3) a batch-corrected expression matrix of the anchors for all cells was obtained and designated as the integrated dataset; 4) the integrated dataset was then normalized again using SCTransform method. Next, the integrated dataset was scaled and “regressed out” on “nCount_RNA” (number of reads per gene) and “percent.mito” (percentage of mitochondria genes per cell) to mitigate the effects of the heterogeneity of these two features on the cell clustering. As for the cell surface protein marker data, the merged ADT dataset was normalized and scaled first using Seurat pre-processing pipeline before being converted to a FlowSet object for batch effects removal using normalizeBatch() function from R Cydar package. The batch-corrected FlowSet object was then switched back to the Seurat ADT dataset to combine with the integrated gene dataset for down-stream analysis.

Cells were then clustered based on gene signals and projected onto the UMAP plot. The cluster of CD57+CD27-CD28-CD244+ CD4+ T cells (Tdiff cluster) were identified by analyzing differentially expressed proteins between clusters. Differentially expressed genes for this target cluster were extracted using the same DE analysis method. Gene set enrichment analysis was performed using R Vision package (18).

R Monocle package was used for Single-cell trajectory pseudotime analysis. Monocle relies on a machine learning technique called reversed graph embedding to construct a learned cell differentiation trajectory that corresponds to the biological process of CD4+ T cells, upon CMV infection, executing through a gene expression program that enables them to transit from being CMV naive to being CMV highly experienced. Next Monocle placed individual cells at their proper position in the trajectory according to their CMV-experienced-ness level defined by the abstract unit of progress called pseudotime (19).

##### VDJ Data

For TCR repertoire analysis, VDJ and CDR3 sequences for the alpha and beta chains of each cell was acquired using cellranger vdj command from python Cell Ranger package. The clonotype for each cell was assigned by cellranger based on their unique combination of paired V(D)J gene usage and paired productive CDR3 sequences of alpha and beta chain. Cells without productive CDR3 sequences or those with more than 1 TRA and 1 TRB were filtered out. There are 25712 cells (out of a total of 34033 cells) that meet the criteria. A data frame was created containing the following columns: barcode of the cell (cell’s identifier), paired V genes, paired J genes, paired CDR3 sequences, clonotype ID and the cell type the corresponding cell belongs to (TN/CM, TEM, Tdiff or Treg). Then the data frame was fed into the sunburstR pipeline to create the sunburst diagram, which is similar to a tree map but with a radial layout for displaying hierarchical structured data. To quantify the differences of clonotype diversity between Tdiff and regular TEM subsets, Simpson’s Reciprocal Index was calculated using R vegan package.

### Statistics and Data Availability

The Wilcoxon signed-rank test was used for the majority of statistical comparisons. For statistical analysis on the Tdiff populations over time in transplant recipients, the R lme4 package was used to perform linear mixed effects regression analysis. The random intercept model (time as fixed, intercept for subjects as random effect) was used rather than the random slope model due to smaller AIC/BIC values *via* ANOVA test. Despite the small sample size, visual inspection of qq residual plots did not reveal obvious deviations from normality. P-values were obtained by likelihood ratio tests of the full model with the effect in question (time) against the null model without the effect (time) in question. All statistical analysis was performed using R programming language. P<0.05 was regarded as statistically different.

Datasets have been uploaded to the Gene Expression Omnibus repository and may be accessed using the accession number GSE167272.

## Results

### CMV Positive Patients Have Increased Populations of Terminally Differentiated CD4+ T Cells in Blood

**Figure 1:**
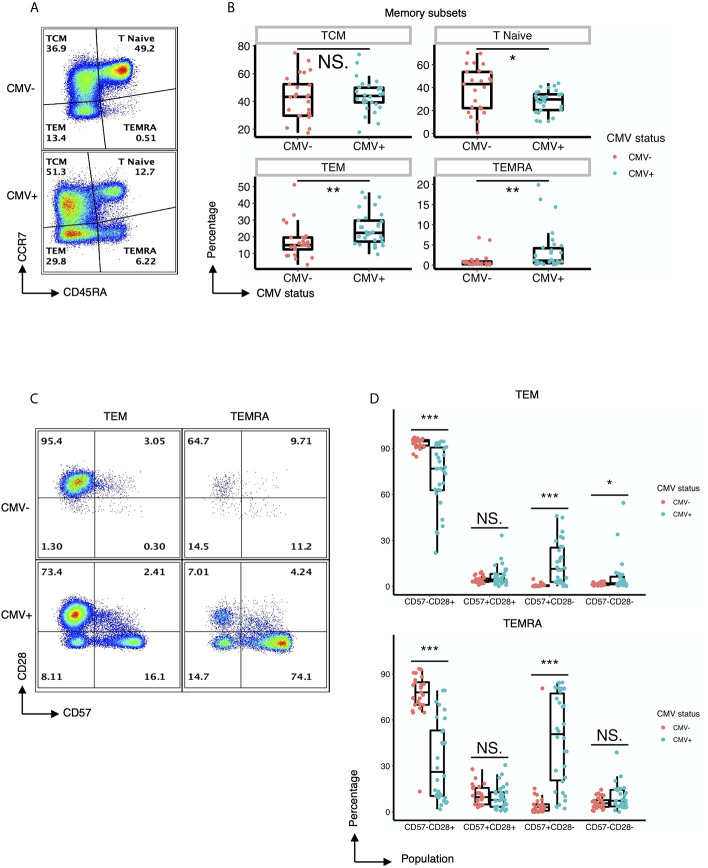
CMV induces expansion of effector memory CD4+ T cell subsets with distinct expression of CD57 and lack of CD28. **(A)** Representative flow cytometry of the TCM, Tnaive, TEM, and TEMRA subsets of CD4+ T cells within CMV- and CMV+ healthy subjects. **(B)** Summary data of the frequency of TCM, Tnaive, TEM, and TEMRA CD4+ T cell subsets in CMV- and CMV+ healthy subjects. Wilcoxon signed-rank Test, ns= nonsignificant, *p<0.05, **p<0.01. **(C)** Representative flow cytometry of the expression of CD57 and CD28 within the TEM and TEMRA subsets of CMV- and CMV+ healthy subjects. **(D)** Summary data of the frequency of CD57-CD28+, CD57+CD28+, CD57+CD28-, and CD57-CD28- subpopulations within the TEM and TEMRA CD4+ T cell subsets in CMV- and CMV+ healthy subjects. Wilcoxon signed-rank Test, ns = nonsignificant, *p<0.05, **p<0.01, ***p<0.001.

As an initial approach to explore the heterogeneity of CD4+ T cells in CMV-infected individuals, we performed traditional user-defined sequential gating on PBMCs derived from 30 CMV seropositive and 25 CMV seronegative subjects to assess the relative abundance of classically defined CD4+ T cell subsets. Despite similar frequencies of the CD45RA-CCR7+ central memory (TCM) cell population, individuals with CMV seropositivity showed a significantly lower frequency of the naïve CD4+ compartment as well as a significant increase in the frequencies of the CD45RA-CCR7- effector memory (TEM) and CD45RA+CCR7- (TEMRA) cell populations (**Figures 1A, B**). These observations were independent of age, suggesting that CMV status was the major driver of the expansion of these subsets of cells (**Supplemental Figure 7**).

**Figure 2:**
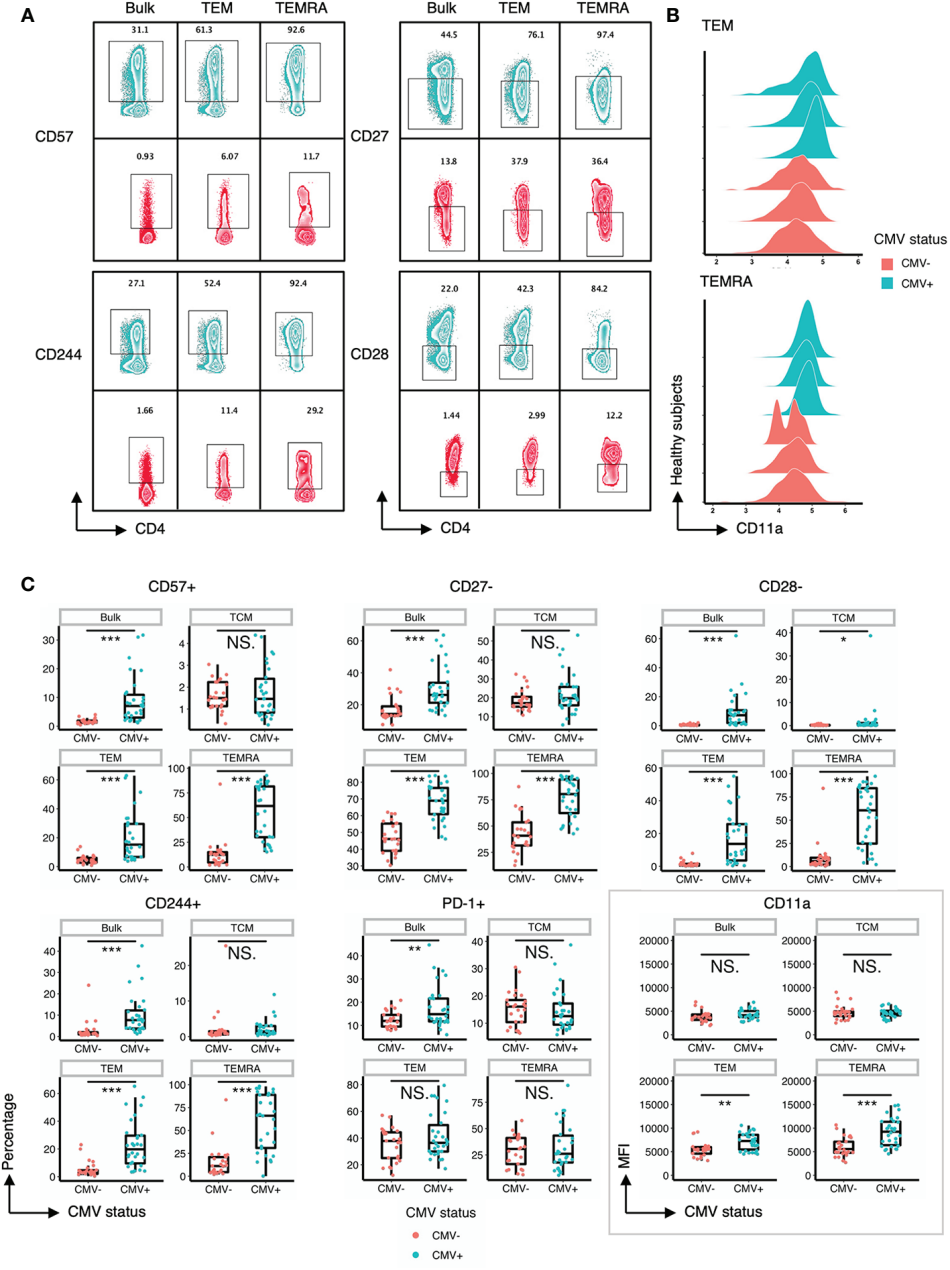
CD57, CD27, CD28, CD244 and CD11a are differentially expressed on T cell subsets within CMV+ and CMV- healthy humans. **(A)** Representative flow cytometry zebra plots of gated CD57+, CD244+, CD27-, and CD28- populations derived from the bulk CD4+ T cells, TEM, and TEMRA subsets within CMV- and CMV+ healthy subjects. Blue= CMV+, Red= CMV-. **(B)** Ridgeline plots of biexponentially transformed MFI of CD11a of CD4+ TEM and CD4+ TEMRA from CMV- and CMV+ healthy subjects. **(C)** Summary data of the frequency of CD57+, CD27-, CD28-, CD244+, and PD-1+ cells, and the biexponentially transformed MFI of CD11a, of the bulk CD4+ T cells, TCM, TEM, and TEMRA subsets within CMV- and CMV+ healthy subjects. Wilcoxin signed-rank Test, ns = nonsignificant, *p<0.05, **p<0.01, ***p<0.001.

Next, we performed a detailed analysis of the phenotype of the CD4+ T cells within CMV+ and CMV- individuals by assessing the expression of costimulatory and coinhibitory molecules, as well as adhesion and senescence-associated molecules, on bulk CD4+ T cells and TEM and TEMRA populations. Bivariate manual gating shows that within the TEM and TEMRA pools in CMV+ subjects, there was a substantial decrease in the frequency of the CD28+CD57- population and a substantial increase in the frequency of CD28-CD57+ and CD28-CD57- cells (**Figures 1C, D**). Detailed analysis revealed that CMV seropositivity was associated with an increase in the frequency of cells expressing the senescent molecule CD57, an increase in the frequency of cells having little to no expression of the costimulatory molecules CD27 and CD28, and an increase in the frequency of cells expressing the co-inhibitor CD244 (**Figures 2A, C**). In addition, there was an increase in the MFI of CD11a (**Figures 2B, C**), but no consistent change in the pattern of PD-1 (**Figure 2C**). These findings indicate the presence of diverse sub-populations of CD4 T-cells in CMV patients as defined by the costimulatory, coinhibitory, and senescence-associated molecules CD27, CD28, and CD57.

### Unsupervised Clustering Loosely Maps to Traditional T Cell Memory Subsets but Reveals More Granular Patterns of CD4+ T Cell Phenotypic Heterogeneity

**Figure 3:**
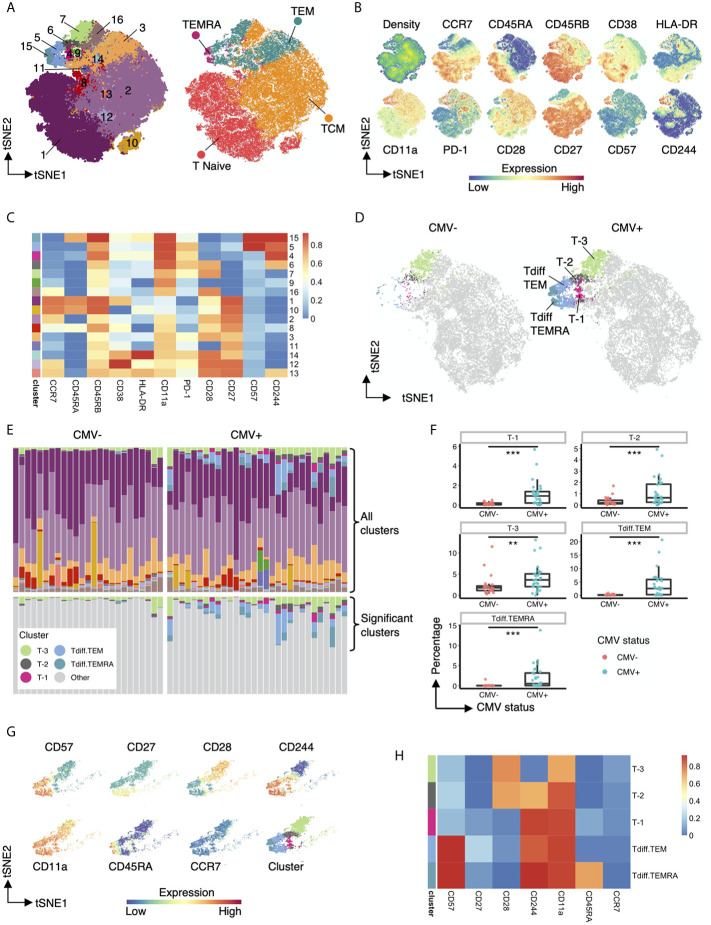
Five heterogenous CD4+ T cell clusters have increased abundance in CMV seropositive individuals. **(A)** Left: tSNE projection of 82,500 concatenated CD4+ T cells sampled equally from CMV+ and CMV- healthy subjects colored by 16 phenotypically distinct clusters identified by FlowSOM. Right: same tSNE projection with cells colored by memory subset membership determined by bimodal gating. **(B)** Same tSNE projection as in **(A)**, with cells colored by the density of cells on the tSNE plot and the expression level of CCR7, CD45RA, CD45RB, CD38, HLA-DR, CD11a, PD-1, CD28, CD27, CD57, and CD244. **(C)** Heatmap of the median marker intensities of CCR7, CD45RA, CD45RB, CD38, HLA-DR, CD11a, PD-1, CD28, CD27, CD57, and CD244 in the 16 CD4+ T cell clusters generated by FlowSOM. **(D)** Same tSNE plot as in **(A)**, but with cells bimodally gated as naive CD4+ T cells removed, and then stratified by CMV status. Clusters not significantly different in abundance between CMV+ and CMV- healthy subjects are grayed out. **(E)** top panel: Relative abundance of the 16 phenotypically distinct populations identified by FlowSOM in each subject, represented by stacked barplot. bottom panel: same stacked barplot as in the top panel but showing only clusters significantly different in abundance between CMV+ and CMV- healthy subjects. **(F)** Summary data of the frequency of the 5 differential clusters in abundance within CMV- and CMV+ healthy subjects. **(G)** Cells that belong to the 5 clusters significantly different in abundance within CMV+ and CMV- healthy subjects are projected onto tSNE space using the same coordinates as in **(A)**, colored by the intensity of expression of CD57, CD27, CD28, CD244, CD11a, CD45RA, and CCR7, and the assigned color for the corresponding cluster as in **(A)**. **(H)** Heatmap of the median marker intensities of CD57, CD27, CD28, CD244, CD11a, CD45RA, and CCR7 in the 5 clusters identified by FlowSOM that are significantly different in abundance between CMV+ and CMV- healthy subjects. Wilcoxin signed-rank Test, **p<0.01, ***p<0.001.

To further characterize the impact of CMV on the CD4 T cell compartment, we performed unsupervised clustering on the flow cytometry data from above. CD4+ T cells from all CMV+ and CMV- study subjects were concatenated and used as input for unsupervised clustering using the flowSOM neural network algorithm (15) to generate self-organizing maps of populations with similar features (**Figure 3**). As an initial step, we compared clusters identified by unsupervised learning (**Figure 3A**, left) with traditional manually gated memory subsets (**Figure 3A**, right) projected onto the same tSNE map. While the clusters loosely mapped with the traditional subsets, there were notable differences. The traditional memory subsets are not contiguous in unsupervised cluster tSNE projection. For example, naive T cells map to cluster 1 but also 10, TCM maps to clusters 2 but also 16, and TEM are found in cluster 3, 5, 6, 7 (**Figure 3A**). Further inspection of the marker expression on the tSNE projection (**Figure 3B**) and in the heatmap of median marker intensity by cluster (**Figure 3C**) provide granular phenotypic information and highlight the heterogeneity of these well-known subsets. For example, naïve T cells as typically gated on parse into two clusters, Cluster 1 and 10, which are distinguished by CD38 expression; and TCM cells parse into 4 distinct clusters (2, 12, 13 and 16), but all with different phenotypes. Several clusters contain two types of T cell subsets, but are differentiated due to high or low expression of specific molecules, i.e. Cluster 9 contains both TCM and TEM but is characterized by low CD27, CD28 and CD244 expression levels.

Next, we compared the differential abundance of CD4+ T cell clusters in CMV+ and CMV- subjects. Visualization of clusters in tSNE space revealed that clusters 4, 5, 6, 7, 15 (from TEM and TEMRA regions) are more prominent in CMV+ subjects (**Figure 3D**). We renamed these clusters to cluster T-3, T-2, T-1, Tdiff.TEM and Tdiff.TEMRA to highlight the late differentiation status of these clusters as defined by their phenotypic state in **Figures 3E–H**. These five CMV-associated clusters are quantitatively larger in CMV+ subjects as shown in the stacked bar (**Figure 3E**) and box and whiskers (**Figure 3F**) plots and demonstrate a unique phenotypic landscape of CD27, CD28, CD244, CD57, CD11a, CCR7 and CD45RA (**Figure 3G**). Interestingly, the most prominent populations in CMV-seropositive individuals, Tdiff.TEM and Tdiff.TEMRA, have high expression of CD57, CD244, and CD11a and low expression of CD27 and CD28. The other three distinct CMV-associated CD4+ T cell populations are similar except T-1 are Tdiff.TEM or Tdiff.TEMRA with low CD57 expression, T-2 are Tdiff.TEM with low CD57 expression and high CD28 expression, and finally T-3 are Tdiff.TEM but low CD57, CD244, and high CD28 expression (**Figure 3H**).

### Transcriptional Features of CMV-Associated CD4+ T Cells

**Figure 4:**
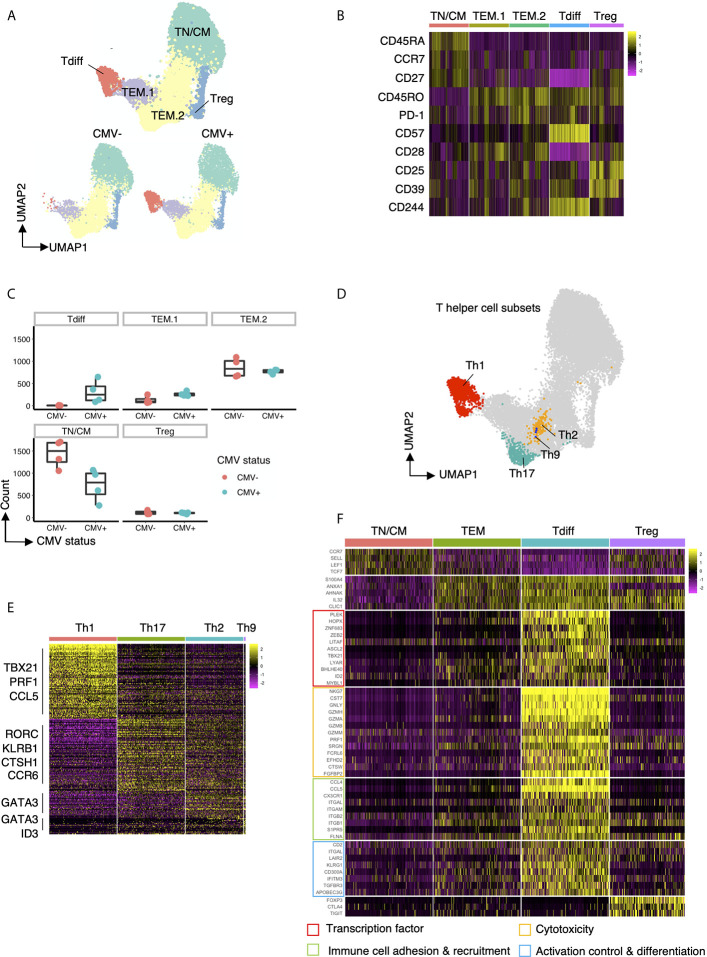
Transcriptomic features of Tdiff cells as defined by their distinct abundance in CMV+ patients. **(A)** top panel: UMAP projection of 15,600 concatenated CD4+ T cells (2,600 cells sampled per subject) colored by the 5 cell types identified based on gene expression. bottom panel: Same UMAP plot as in the top panel, stratified by CMV status. **(B)** Heatmap of differentially expressed cell surface protein markers for each cluster. **(C)** Summary data of the cell abundance in each of the five major subtypes within CMV- and CMV+ healthy subjects. **(D)** Same UMAP projection as in **(A)** with cells colored by T helper cell types. **(E)** Heatmap of the differentially expressed genes for each T helper cell type (Th1, Th17, Th2, Th9). **(F)** Heatmap of the differentially expressed genes for each major cell type (TN/CM, TEM, Tdiff, and Treg) categorized into four major groups based on function: (1) transcription factors; (2) genes associated with cytotoxicity; (3) genes associated with immune cell adhesion and recruitment; and (4) genes associated with activation control (genes coding for co-inhibitory and co-stimulatory molecules) and differentiation.

To further investigate the properties of CD4+ clusters T cells that are enriched in CMV+ healthy subjects, we performed single-cell RNAseq on CD4+ cells isolated from CMV+ and CMV- patients. Using the Seurat louvain clustering algorithm, we identified 5 distinct clusters from these individuals (**Figure 4A**). Exploration of the top differentially expressed surface protein markers and genes (**Figures 4B, F**) for each cluster revealed a naive T cell and/or TCM cluster due to the upregulation of CD45RA, CD27, and CCR7, confirmed *via* differential gene expression of *CCR7*, *SELL* (L-selectin), *LEF1* (20) and *TCF7* (21). We further identified a cluster of Tregs with high protein expression of CD25 and CD39 as well as increased transcript expression of *FOXP3*, *CTLA4*, and *TIGIT*. Interestingly, we identified three clusters of effector memory T cells, labelled as TEM.1, TEM.2, and Tdiff, based on high expression of *S100A4*, *ANXA1*, *AHNAK*, *IL32*, *CLIC1*, genes all shown to be enriched in effector memory T cells (22, 23). The relative abundance of clusters in CMV seropositive and seronegative individuals reveal that cells within the “Tdiff” cluster almost exclusively exist in the CMV seropositive individuals (**Figures 4A, C**). Of note, surface protein analysis further show that while TEM.1 and TEM.2 are CD57-, cells in cluster Tdiff are distinctly CD57+, CD27-, CD28-, and CD244+, similar to the late differentiation Tdiff.TEM an Tdiff.TEMRA populations identified in the flow cytometric analyses in **Figure 3H** (**Figure 4B**). Assessment of T-helper subset phenotype revealed that the Tdiff cluster is predominantly Th1 cells due to their high expression of *TBX21*, *PRF1*, and *CCL5* (**Figures 4D, E**). To identify the other T helper subsets within the TEM.1 and TEM.2 clusters, we reclustered cells and identified Th17, Th2, and Th9 *via* known transcription factors and projected those subsets on the UMAP (**Figure 4D**). These data identified Th2, Th9, and Th17 cells localizing to the TEM.2 cluster.

We further assessed the transcriptomic profile of the CD57+ Tdiff cells by comparing it to the transcriptomic profile of CD57- effector memory T cells, naive/TCM, and Tregs (**Figure 4F**). Differential expression analysis revealed 140 transcripts with significantly higher mean expression by at least 2-fold (with maximum P value 9.07E-134) in single cells from CD57+CD27-CD28-CD244+ T cells compared with naïve/TCM, CD57- effector memory T cells, and Tregs (**Supplemental Table 1**). Many upregulated transcripts in the CD57+ Tdiff cluster were cytotoxic genes that are preferentially expressed in CD8+ T cells and NK cells, including CD8+ T cells and NK cells signature genes such as *NKG7*, *CST7*, *GNLY*, *GZMH*, *GZMA*, *GZMB*, *GZMM*, *PRF1* and other cytotoxicity-linked transcripts (**Figure 4F**), The Tdiff cluster also expressed high levels of integrins that mediate cell-cell adhesion during cell recruitment to sites of inflammation (24), including *ITGAL* (encodes CD11a), *ITGAM* and *ITGB2* (encodes CD11b) which pair as “Mac-1”, and *ITGB1* (encodes the CD29 beta 1 subunit involved in the formation of VLA4 and VLA5). Distinguishing chemokines and chemokine receptors, *CCL4*, *CCL5* and *CX3CR1* (fractalkine receptor) were highly differentially expressed (at least 2.8-fold) relative to the other populations. Other genes involved in leukocyte trafficking that were differentially expressed in Tdiff include *S1PR5*, *FLNA* (25, 26) and *SPON2* (27).

In addition to cytotoxic-associated and trafficking-associated molecules, Tdiff express a distinct complement of co-stimulatory, co-inhibitory, and signaling adhesion molecules that are known to tune the threshold of T cell activation (**Figure 4F**). At the protein level they show reduced expression of CD28 and CD27, but increased LFA-1. At the transcript level Tdiff shows significantly increased *CD2* expression, as well as the genes that make up CD11a and VLA4, all of which deliver signals that contribute to T cell activation. These cells also express high transcript levels of *LAIR2* (*CD306*), a soluble receptor that blocks signaling *via* LAIR1, an ITIM containing co-inhibitory molecule (28). In addition, Tdiff differentially express the genes encoding the co-inhibitory receptors KLRG1 and CD300A, an ITIM-containing molecule involved in the recognition phosphatidylcholine and phosphatidylserine on dying cells. Notably other coinhibitory genes not differentially expressed included *CTLA4*, *PD1*, *TIGIT*.

Analysis of transcription factor expression revealed a distinct profile that maps to the properties of Tdiff as a persistent, patrolling population of memory cells that is maintained in large numbers in peripheral blood in a state that is poised for cytotoxicity (**Figure 4F**). Transcription factors *ZEB2* (29, 30), *ID2* (31), *ZNF683* (32, 33), *PLEK* (32), and *TBX21* (T-bet) (29, 30) are linked to T cell cytotoxic function and differentiation. Tdiff also express other transcription factors that regulate cell growth, cell cycle progression, differentiation and apoptosis: *LITAF*; *MYBL1* (34); *LYAR*; *HOPX* (35); *ASCL2*, a transcription factor shown to promote Tfh and is regulated by *ID2* (36, 37); and *BHLHE40* (38), a transcription factor implicated in the maintenance of effector & memory CD8+ TRM and tumor-infiltrating lymphocyte (TIL) fitness and functionality (39, 40).

### Repertoire Analysis Reveals Striking Oligoclonality of the CD4+ CMV-Associated T Cell Subset

**Figure 5:**
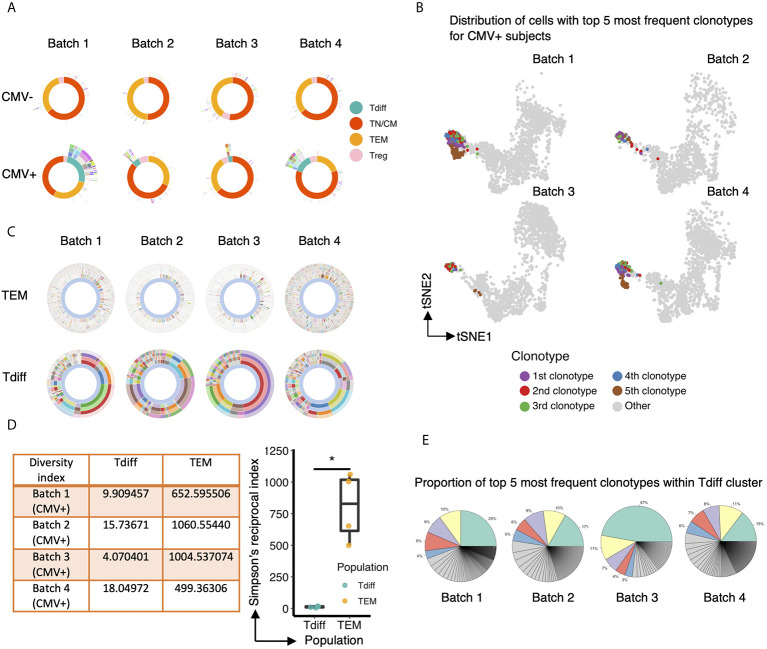
The TCR repertoire of the CD4+ T cell subset enriched in CMV+ individuals is markedly oligoclonal compared to the other T cell subsets. **(A)** Sunburst plots to compare the overall TCR clonotype diversity in CMV- and CMV+ healthy humans in each batch. Each batch includes TCR repertoire data from one seropositive and one seronegative individual, age and gender matched. The sunburst plot illustrates the hierarchical structure of an individual’s TCR repertoire data. Each sunburst plot consists of 5 concentric circles. The center of the circle represents the membership of the gene cluster to which each cell is assigned. Each color block stands for a different gene cluster. The second, third and fourth layer reflects the paired V genes, paired J genes, and paired CDR3 sequences, respectively, adopted by the corresponding cell. The outermost circle represents the clonotype of each corresponding cell. Colors represent a unique V, J, CDR3, or clonotype, with the conglomeration of many colors resulting in the appearance of white. **(B)** Same UMAP projection as **Figure 4A** is shown for each CMV+ subject, with cells containing the top 5 most frequent clonotypes colored by their clonotype membership. **(C)** Comparison of TCR clonotype diversity between CD57- effector memory T cells (top row) and Tdiff cells (bottom row) in each CMV+ healthy human, represented by sunburst plots similar to **(A)**. Colors represent a unique V, J, CDR3 sequence, or clonotype (as in A), with the conglomeration of many colors resulting in the appearance of white. **(D)** Left: Summary table of the Simpson’s Reciprocal Index of Tdiff and TEM subsets for each CMV+ healthy subject. Right: Summary data of the Simpson’s Reciprocal Index of Tdiff and TEM subsets for each CMV+ healthy subject, Wilcoxon signed-rank Test, *p<0.05. **(E)** Proportion of the top 5 most frequent clonotypes within the Tdiff cluster for each CMV+ subject. Wilcoxin signed-rank Test, *p<0.05.

Next, we explored the characteristics of the TCR repertoire of the CD4+ Tdiff and how these TCRs were distributed across the entire CD4+ pool of cells. All subsets in CMV negative subjects showed a high level of diversity without evidence of significant clonal expansion in any subset (**Figure 5A**, top panel). In contrast, in each of the CMV+ samples, prominent clonal expansion was readily apparent, particularly within the Tdiff cell subset (**Figure 5A**, bottom panel). Mapping the top 5 most frequent clonotypes onto the tSNE projection revealed that all top 5 clones were almost exclusively in the Tdiff cluster (**Figure 5B**). Further comparison of the TEM vs Tdiff clonal diversity confirmed markedly reduced diversity in the Tdiff cells as compared to TEM cells in the CMV+ subjects (**Figure 5C**, top and bottom, respectively). Using Simpson’s Reciprocal Index as a measure of TCR clonotype diversity, we quantified the marked oligoclonality of Tdiff relative to the highly diverse TEM population (mean, 11.9 vs 733.5, p <0.05) (**Figure 5D**). Analysis of the proportion of clonotypes within the Tdiff subset revealed that the top 5 most common clonotypes can take up 47-72% of the whole TCR repertoire (**Figure 5E**). These data indicate that the TCRs in the Tdiff cluster were derived from a very narrow range of receptor specificities, suggesting that these cells are driven by exposure to a specific antigen. In addition, these TCRs were minimally found in other clusters, suggesting that specificity for these antigens could drive this cytotoxic fate.

As the oligoclonality of Tdiff suggest similar specificities, we interrogated whether Tdiff preferentially respond to CMV antigens. To do this, we used CD57 as a surrogate marker for Tdiff, as we show in **Figure 4B** the clear upregulation of CD57 in Tdiff compared with the TEM.1, TEM.2, Treg, and TN/TCM subsets. Following an 8-hour stimulation of healthy CMV+ PBMC with CMV peptides derived from pp65 and IEL, we found a significantly increased frequency of TNF+IFN*γ*+ cytokine producers in the CD4+ CD57+ T cell subset compared to the CD4+ CD57- subset, and compared to unstimulated controls (**Supplemental Figure 8**). These data provide evidence that Tdiff harbors a small fraction of CMV peptide-responsive T cells, emphasizing that while some Tdiff are capable of responding to CMV antigens, the bulk of Tdiff are unable to respond.

### The Frequency of CD4+CD57+ TEM and TEMRA Significantly Increases Overtime in Transplant Recipients on Belatacept Therapy

Due to the marked clonal expansion of CD4+ T cells within individuals, we hypothesized that individuals with CMV would incur an increase in Tdiff-like populations over time. To assess whether the frequencies of CD4+CD57+ TEM and CD4+CD57+ TEMRA subsets change over time, we collected blood samples at 6-month intervals from healthy, CMV+ individuals (**Supplemental Figure 9A**). In addition, we performed an exploratory analysis of samples at 2-month intervals from stable, CMV+ renal transplant recipients on the CD28 costimulation blocker, belatacept (**Supplemental Figure 9B**). We found that time affected the frequency of CD4+CD57+ TEM subset (χ2(1)=5.58, p=0.01817), increasing it by about 2.72% ± 1.11 (SE), and affected the frequency of CD4+CD57+ TEMRA subset (χ2(1)=8.08, p=0.004484), increasing it by about 1.93% ± 0.62 (SE) in renal transplant recipients at the interval assessed, but these differences were not evident in healthy individuals (**Supplemental Figure 9A, B**). Renal transplant recipients on belatacept represent a unique population of individuals without productive signaling through CD28. With the specific blockade of CD28+ T cells, CD28- T cells are left unhindered. Because Tdiff have been defined here as CD28- and CD57+, belatacept could represent a specialized circumstance in which CD28- Tdiff cells are allowed to expand. Confirming this, the overall proportion of CD57+ TEM and TEMRA subsets are elevated in belatacept patients (**Supplemental Figure 9C**), suggesting a relatively rapid increase in the proportion of TEM and TEMRA cells that express CD57+. Although the timeframe assessed for each individual recipient is short, these data suggest that there is a continual, progressive expansion of Tdiff cells in CMV seropositive individuals that is not suppressed *via* CD28 T-cell targeted immunosuppression.

### Single Cell Trajectory Analysis of CMV-Associated CD4+ T Cell Clusters Reveals Progressive Differentiation Program

**Figure 6:**
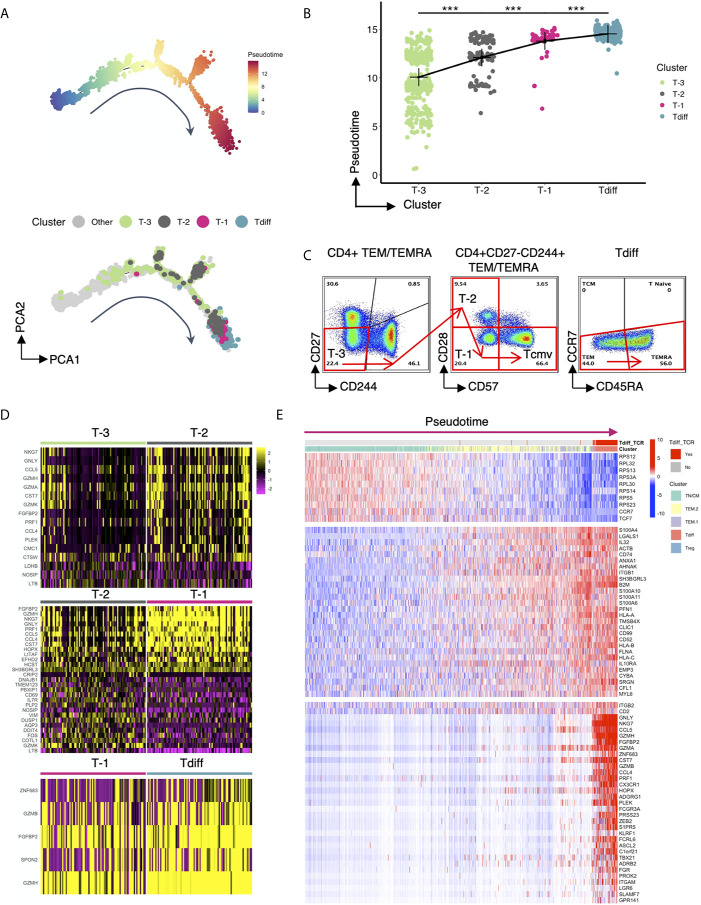
Single cell trajectory analysis of CMV-associated CD4+ T cell clusters reveals progressive differentiation program. **(A)** Top panel: the single-cell trajectory reconstructed by Monocle representing the hypothesized biological process of CD4+ T cells undergoing transcriptional reprogramming after CMV exposure. Cells are ordered and colored by pseudotime, an abstract unit of progress along the trajectory. Bottom panel: Same trajectory as in the top panel, with CMV-associated phenotypes (cluster T-3, T-2, T-1 and Tdiff) shown in their assigned colors as in **Figure 3D**. Cluster T-3, T-2, T-1 and Tdiff were manually gated based on the cell surface protein expression matrix derived from the ADT library of single cell RNAseq data using R CytoRSuite package. **(B)** The order of clusters T-3, T-2, T-1, Tdiff is arranged based on the significant gradual increase in their median pseudotime level, represented by a summary dot and line plot. **(C)** Flow cytometry plots showing the proposed gating strategy based on pseudotime for CMV-associated phenotypes. **(D)** Heatmaps showing differentially expressed genes that distinguish cells of cluster T-3 from cluster T-2, cluster T-2 from cluster T-1, and cluster T-1 from cluster Tdiff. **(E)** Heatmap of the expression of 3 groups of genes as a function of pseudotime level. The expression level of these genes variate most significantly with pseudotime compared to others. Cells are ordered by pseudotime and colored by their gene cluster membership as in **Figure 4A** and whether the cells contain TCR clonotypes that are within the Tdiff cluster (labeled as Tdiff_TCR). Wilcoxin signed-rank Test, ***p<0.001.

To further understand how this unique, oligoclonal population of cytotoxic CD4+ T cells might form in CMV+ patients, we performed single-cell trajectory analysis of the transcriptomic data to explore how the genetic profile of Tdiff is acquired. Ten-thousand cells were downsampled for analysis. A single-cell differentiation trajectory was constructed based on transcriptomes, representing the hypothesized biological process of CD4+ T cells going through genetic reprogramming after CMV exposure. Cells were placed onto the trajectory in the order of pseudotime (19), a measure of cells’ progress through the transition (**Figure 6A**, top panel). CD4+ T cells with CMV-associated phenotypes (phenotypes of cluster T-3, T-2, T-1 and Tdiff based on **Figure 3D**) were manually gated *via* R package CytoRsuite based on cell surface antibody barcodes and were shown on the trajectory (**Figure 6A**, bottom panel) in their assigned colors as in **Figure 3D**. We observed that CD4+ T cells undergo transcriptomic changes to become cells of cluster T-3, T-2, T-1 and Tdiff sequentially in the differentiation process (**Figure 6A**), which is further supported by statistical comparison of pseudotime values among these 4 clusters (**Figure 6B**). The phenotypical changes during the differentiation process for CMV-induced memory CD4+ T cells is illustrated by representative flow plots shown in **Figure 6C**.

To understand the gene regulation program that facilitates the progressive stepwise phenotypical changes within CMV-associated clusters, the differentially expressed genes that distinguish cluster T-3 from cluster T-2, cluster T-2 from cluster T-1 and cluster T-1 from cluster Tdiff were analyzed and presented in a heatmap (**Figure 6D**). Based on our findings, we hypothesized that the infection of CMV incites extensive memory inflation and a specific differentiation course of CD4+ T cells: first, CD27- effector memory CD4+ T cells that express adhesion molecule CD11a (T-3) are expanded. As differentiation progresses, cytotoxicity-related transcription factor *PLEK* upregulates cytotoxicity-related genes such as *NKG7*, *GNLY*, *CCL5*, *CCL4*, *CST7*, *PRF1*, *GZMH*, *FGFBP2* etc. These cells then gain the co-inhibitory molecule CD244 upon conversion to the T-2 differentiation state. Notably, T-2 does not have higher PD-1 or CTLA-4 expression level, suggesting that they might not assume a classic exhaustion state. Next, T-2 loses primary costimulatory receptor CD28 to differentiate into T-1. This step is accompanied by upregulation of cytotoxicity-related genes including *EFHD2* and is governed by transcription factor *HOPX*, a T cell plasticity regulator important for the survival and persistence of Th1 effector/memory cells (35), and transcription factor *LITAF*, a p53-inducible gene tumor suppressor. Finally, T-2 transforms to T-1 through acquisition of the “replicative senescence” marker CD57, which is stimulated by cytotoxicity-related genes *GZMB*, *FGFBP2*, *GZMH*; cytotoxicity-related transcription factor *ZNF683* and *SPON2*, a gene involved in leukocyte trafficking. As can be seen in **Figure 6D**, transcriptomic changes from T-2 to T-1 is most evident, suggesting that the transcriptional regulation leading to the loss of CD28 might be the most crucial step during the reprogramming process.

To confirm that the regulating genes mentioned above that promote the formation of Tdiff cells are turned on at the very end of the constructed biological process, genes that variate in the expression level most significantly with pseudotime were selected and presented in the heatmap (**Figure 6E**). In this heatmap cells were ordered by pseudotime from left to right and colored by the gene cluster membership as in **Figure 4A** and whether the cells contain TCR clonotypes that ever appear in the cells of the Tdiff cluster (labeled as Tdiff_TCR). The genes in this heatmap were grouped based on the variation patterns. As can be seen in the figure, throughout the differentiation process, genes that were specific to TN/CM, such as genes in the RPS family, *CCR7*, and *TCF7*, were gradually lost whereas genes specific to TEM, such as *S100A4*, *IL32*, *ANXA*, *AHNAK* etc., were gained gradually (**Figure 6E**). Genes that were acquired at the end of the pseudotime spectrum are composed of almost all differentially expressed genes for Tdiff subset, including transcription factors (*PLEK*, *HOPX*, *ZNF683*) and cytotoxicity-related genes (*GNLY*, *NKG7*, *CCL5*, *GZMH*, *FGFBP2*, *GZMA*, *CST7*, *GZMB*, *CCL4*, *PRF1* etc.) that participate in the differentiation of CMV-associated CD4+ T cells.

### T Cell Subsets Previously Identified in Transplant Rejection Associate With the Tdiff Population

**Figure 7:**
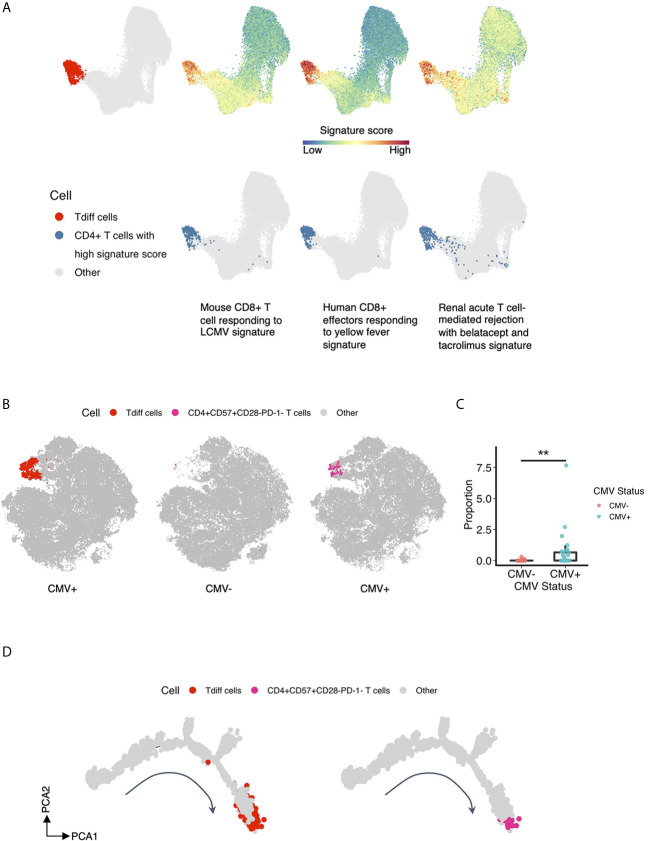
T cell subsets previously identified in rejection associate with the Tdiff population. **(A)** Gene set enrichment analysis was performed of the five T cell subsets identified in **Figure 4A** to gene sets involved with (1) mouse CD8+ T cells responding to LCMV, (2) human CD8+ effectors responding to yellow fever, and (3) renal acute T cell-mediated rejection with belatacept and tacrolimus. The first column showcases the reference Tdiff cells colored in red and projected onto the same UMAP map as in **Figure 4A**. For the rest of the columns, the first row showcases the same UMAP projection as in **Figure 4A**, with cells colored by its signature score value for each gene set, whereas in the second row, cells with high signature score (at more than 97th percentile) for each gene set were highlighted and shown in blue. **(B)** The first column showcases the same tSNE projection as in **Figure 3A**, with manually gated Tdiff cells from the flow cytometry data using the R CytoRSuite package colored in red. The second and third column showcase the same tSNE projection as in **Figure 3A**, with manually gated CD4+CD57+CD28-PD-1- T cells from the flow cytometry data using the R CytoRSuite package colored in deep pink and stratified by CMV status. Other types of cells are all colored in gray. **(C)** Statistical comparison of the frequency of the manually gated CD4+CD57+CD28-PD-1- T cell subset between CMV+ and CMV- healthy subjects, represented by a summary box and dot plot. **(D)** Shown on the left is the same single cell trajectory as in **Figure 6A**, with Tdiff cells colored in red and other cells in gray. Tdiff cells were manually gated from the cell surface protein expression matrix derived from the ADT library of single cell RNAseq data using R CytoRSuite package; whereas shown on the right is the same single cell trajectory as in **Figure 6A** as well, with CD4+CD57+CD28-PD-1- T cells colored in deep pink and other cells in gray. CD4+CD57+CD28-PD-1- T cells were also manually gated from the cell surface protein expression matrix derived from the ADT library of single cell RNAseq data using R CytoRSuite package. Wilcoxin signed-rank Test, **p<0.01.

Recent evidence from Espinosa et al. suggests that a similar phenotype of CD4+ T cells as to what is presented here are associated with costimulation blockade-resistant rejection. To garner a more complete understanding of the function of this population as cytotoxic effectors, and potentially their role in transplant rejection, we performed gene set enrichment analysis of the CD4+ T cells analyzed by single-cell RNA sequencing and compared them to canonical activated CD8+ T cell gene signatures—antigen-specific human CD8+ T cell effectors at the peak of response to yellow fever and mouse CD8+ T cells responding to LCMV—and to a gene set generated based on genes correlated with T cell-mediated rejection in patients treated with belatacept or tacrolimus (41). CD4+ T cells enriched in expression of genes within each gene set were defined by a signature score (18) and projected onto the same tSNE map as shown in **Figure 4A** and presented in **Figure 7A**, 2^nd^ to 4^th^ column in the top panel. CD4+ T cells at or above the 97^th^ percentile in signature score are highlighted in blue in **Figure 7A**, bottom panel. We observed that Tdiff cells were enriched in genes associated with virus-specific CD8+ T cells, suggesting these cells are transcriptionally similar to cytotoxic CD8+ T cells. We further found that Tdiff cells were highly enriched in genes associated with rejection. Together, these data suggest that the Tdiff cells take on a transcriptional program similar cytotoxic CD8+ T cells and that this cytotoxic CD4+ T cell program is enriched in phenotypes capable of eliciting organ rejection.

Finally, we explored the similarity of the CD4+ Tdiff identified here to the CD57+CD28-PD-1- CD4+ T cell subset that is associated with increased rates of rejection in patients treated with the CD28 blockade therapeutic belatacept (42). To do this, we manually gated on this subset of cells from the flow cytometry data using the R CytoRSuite package and projected them onto the tSNE plot shown in **Figure 3A** (**Figure 7B**). Results indicate that this subset is concentrated within the Tdiff cluster (as shown in **Figure 3D**) and is significantly enriched in CMV+ individuals (**Figure 7C**), confirming the transcriptomic similarities in rejection-associated T cell populations and Tdiff. To assess whether this rejection-associated subset also populated a similar location in the pseudotime analysis as the Tdiff, we manually gated on this subset of cells from the cell surface protein expression matrix derived from the ADT library of single cell RNAseq data using the R CytoRSuite package and projected them onto the differentiation trajectory shown in **Figure 6A** (**Figure 7D**). We found that the rejection subset aligns with the Tdiff subset on the differentiation trajectory, suggesting these populations are similar in differentiation.

## Discussion

CMV is among the most dominant influences on the T cell compartment of healthy humans (1). Because CMV exposure alters a major arm of adaptive immunity, CMV has implications in individuals with infections, cardiovascular disease, increased age, cancer, and transplanted organs and remains an important area of study. By integrating emerging analytic approaches, i.e. unsupervised clustering, trajectory analysis, and single-cell multi-omics data, we provide new insight into the heterogeneity and differentiation of these large oligoclonal populations of CD4+ T cells that are retained in healthy CMV seropositive individuals. In brief, we find CMV seropositive individuals retain multiple populations of CD4+ T cells that exhibit stepwise loss of costimulatory molecules, gain of cytotoxic function, and gain of coinhibitory molecules. Our findings using traditional user-defined gating of high dimensional flow cytometry data are consistent with prior observations that CMV seropositivity is tightly correlated with the frequency of CD4+, CD28dim, CD57+, CD244+ T cells (43–45). Importantly, transcriptomic assessment of these populations revealed tight associations with effector CD8+ T cells and with transplant rejection, highlighting the need for further studies in understanding the role and function of these cells following CMV infection.

Here, transcriptomic analysis of CMV-induced CD4+ T cells revealed the specific retention of TEM and TEMRA CD4+ T cells that maintain cytotoxic function, named here for their late differentiation status as Tdiff. Other persistent viral infections such as Dengue and Hepatitis C have also been shown to drive the differentiation of cytotoxic CD4+ T cells with a profile that does not fit neatly into the Th1/2/9/17 paradigm (32, 46, 47). Despite close alignment with the Th1 subset due to upregulation of genes like *TBX21*, *PRF1*, and *CCR5*, Tdiff set themselves apart by their cytotoxic signature found to be associated with viral-induced effector CD8+ T cells. Studies in mice suggest that CD4+ CTLs are important for control of CMV, and their development predicts protection after vaccination (9). Despite the large focus on Th1-mediated protective immunity, these studies and our study showing the specific retention of cytotoxic CD4+ T cells in CMV seropositive individuals suggest the importance of understanding the role of these CTL-like CD4+ T cell populations in CMV protective immunity.

We further show through single cell trajectory analysis that obtaining cytotoxic function precedes the upregulation of CD57 and follows the loss of CD28. Although CD57 has typically been considered a coinhibitory molecule associated with senescence (48, 49), work also describes the association of CD57 with cytotoxicity (42, 50–52). Our finding provides further clarification of the link between CD57 and cytotoxicity on CD4+ T cells, implying that there is a specific, stepwise differentiation trajectory in which cells lose CD28, then gain cytotoxicity, and then gain CD57. This specific trajectory makes it plausible that cytotoxic CD4+ T cells gain CD57 as a means to regulate the activation of these potent, cytolytic cells.

In addition to the loss of the costimulatory molecules CD27 and CD28, we find that these cells have significantly increased transcripts for *CD2*. CD2 has recently been implicated as having costimulatory potential in CD8+ T cells that lack CD28 (53), suggesting it as an alternative pathway to canonical costimulation. In these Tdiff subsets, CD2 could have the same function and provide some level of costimulation despite loss of CD27 and CD28.

Tissue-resident memory T cells have recently been identified in several peripheral sites in individuals with CMV (54), suggesting an important involvement of Trm in the response against CMV. Although only peripheral blood samples were assessed in this study, increased expression of transcripts for *CX3CR1*, several integrins, and the trafficking receptor *S1PR5* suggests enhanced ability to enter inflammatory sites. Whether Tdiff are maintained in peripheral tissues, however, as tissue-resident cells, is uncertain, but evidence presented here describe the likelihood of their ability to traffic into tissue and elicit effector function.

Another distinctive feature of these CMV-induced Tdiff CD4+ T cells is their marked oligoclonality, suggesting that this expansive and cytolytic population of T cells was derived from only a handful of clones as compared to the TN/TCM, TEM, and Treg subsets. These data imply that these cells arise from a narrow range of specificities and could be somewhat similar to inflationary CMV-specific CD8+ T cells with regards to their continual presence and expansion overtime. Inflationary CMV-induced CD4+ T cells have been previously described in mice (55), but robust evidence for this phenomenon is lacking. Our data suggest that the Tdiff-induced CD4+ T cell population is inflationary due to their limited diversity; however, further studies are warranted to confirm this finding.

Interestingly, the cytotoxic CD244+CD28-CD57+ CD4+ Tdiff subset that we have identified in CMV-seropositive individuals here has also been identified in many other disease states as “risky” T cells subsets (42, 50–52); however, the origin of this population in these disease states is unknown. We found that the gene signature of CMV-associated Tdiff cells had a significant association with gene signatures of effector CD8+ T cells generated from yellow fever vaccine and of T cell mediated rejection following transplantation. This analysis suggests that this CMV-induced subset of CD4+ T cells is highly robust and CD8+ T cell-like, as evidenced by our assessment of their cytotoxicity, and has the capability of causing rejection. Interestingly, a similar population of CD4+ T cells (CD57+CD28-) was found to increase upon CMV infection in renal transplant recipients (44) and is associated with costimulation blockade-resistant rejection (CoBRR) (42). Our data indicate that Tdiff and this subset of risky T cells in transplant recipients are similar, indicating that Tdiff possess a phenotype that could potentiate risk of rejection. As such, future studies will interrogate whether transplant recipients harbor CMV-induced T cell populations that can contribute to rejection.

Here, we have shown a clear enrichment of particular CD4+ T cell subsets in CMV-seropositive individuals. We have further provided evidence that only a small subset of these enriched cells are able to respond to CMV antigens, although significantly elevated compared to the non-enriched subsets. Whether non-responsive Tdiff cells are enriched due to conserved immune-environmental changes that occurs with infection (i.e. cytokine milieu, microenvironmental pressures, etc.) that induces bystander activation, or whether they are specific to CMV but non-responsive due to coinhibitory and checkpoint molecules, remains to be determined. Despite the unknown, we suspect that these induced subsets have long-term effects on the immune response in other disease states like transplant rejection, as shown by the phenotypic similarities of Tdiff with risky T cell subsets in transplant recipients and the specific expansion of these subsets overtime in transplant recipients on immunosuppression. Future studies undertaken will seek to understand the factors that lead to the specific enrichment of these subsets in CMV-seropositive individuals.

Using detailed approaches to analyze the CD4+ T cell compartment in CMV-seropositive and -seronegative individuals, we provide new insight into the differentiation and transcriptome of the heterogeneous populations induced following CMV infection. Because we found that the transcriptome of these CMV-induced subsets is associated with T cell mediated rejection, we propose future studies of these cells to determine their impact on subsequent disease states. Increased knowledge of their helpful or harmful roles can inform targeted strategies to manipulate these populations for better outcomes.

## Data Availability Statement

The datasets presented in this study can be found in online repositories. The names of the repository/repositories and accession numbers can be found below: Gene Expression Omnibus, GSE167272.

## Ethics Statement

The studies involving human participants were reviewed and approved by Emory University Institutional Review Board. The patients/participants provided their written informed consent to participate in this study.

## Author Contributions

WZ and CPL designed the study. ABM, WZ, HTK, and CPL wrote the manuscript. EVP and JMR collected data. WZ, GK, HTK, and CPL analyzed the data. All authors edited the manuscript. All authors contributed to the article and approved the submitted version.

## Funding

This work was supported by the James M. Cox Foundation, the Carlos and Marguerite Mason Trust, and NIH 1U01AI138909-01, awarded to CPL.

## Conflict of Interest

The authors declare that the research was conducted in the absence of any commercial or financial relationships that could be construed as a potential conflict of interest.
