# Supplementary material for: CMV Status Drives Distinct Trajectories of CD4+ T Cell Differentiation
Source: Front Immunol. 2021 Apr 15;12:620386. doi: 10.3389/fimmu.2021.620386 (PMC8081907; doi:10.3389/fimmu.2021.620386)
Supplement: Supplementary file 1 [file DataSheet_1.docx]

***Supplementary Material***

**1 Supplemental Figures**

**Supplemental Figure 1**

**Supplemental Figure 1:** **Experimental and analytic workflow.** Fresh PBMCs from 30 CMV+ and 25 CMV- healthy subjects were stained with the following antibodies: CD3, CD4, CD8, CD45RA, CCR7, CD57, CD27, CD28, CD244, CD11a, PD-1, HLA-DR, CD38, CD45RB and Live/Dead Aqua. Flow files were acquired and QCed. First, manual sequential gating in FlowJo was carried out to determine markers of interest and assess CD4+ T cell subpopulation abundance. Then, unsupervised clustering of high dimensional flow cytometry data was conducted to explore interaction among markers of interest and find novel phenotypes. CD4+ T cells were isolated through autogating R package openCyto and randomly sampled in FlowJo to 5000 cells/subject. Self-organizing map R FlowSOM package was used to cluster concatenated CD4+ T cells into phenotypically different populations which were then projected onto tSNE space using RtSNE. To understand the transcriptome changes induced by CMV, four pairs of age- and sex-matched CMV-seropositive and CMV-seronegative healthy humans from the flow cytometry cohort were selected for single cell multi-omics sequencing. CD4+ T cells from fresh PBMCs were extracted using MACS negative CD4+ T cell isolation kit and stained with the following TotalSeq type C feature barcode antibodies (Biolegend): CD57, CD27, CD28, CD244, CD45RA, CCR7, CD45RO, PD-1, CD25 and CD39. RNA, ADT and V(D)J library preparation and sequencing were completed using 10X Genomics workflow. Fastq files, expression matrix and TCR clonotype data were generated by Python CellRanger package. Cell clustering based on gene signals with differential expression analysis using Seurat package, gene set enrichment analysis using VISION package, TCR repertoire analysis using sunburstR and vegan package and Pseudotime analysis using Monocle package were then performed.

**
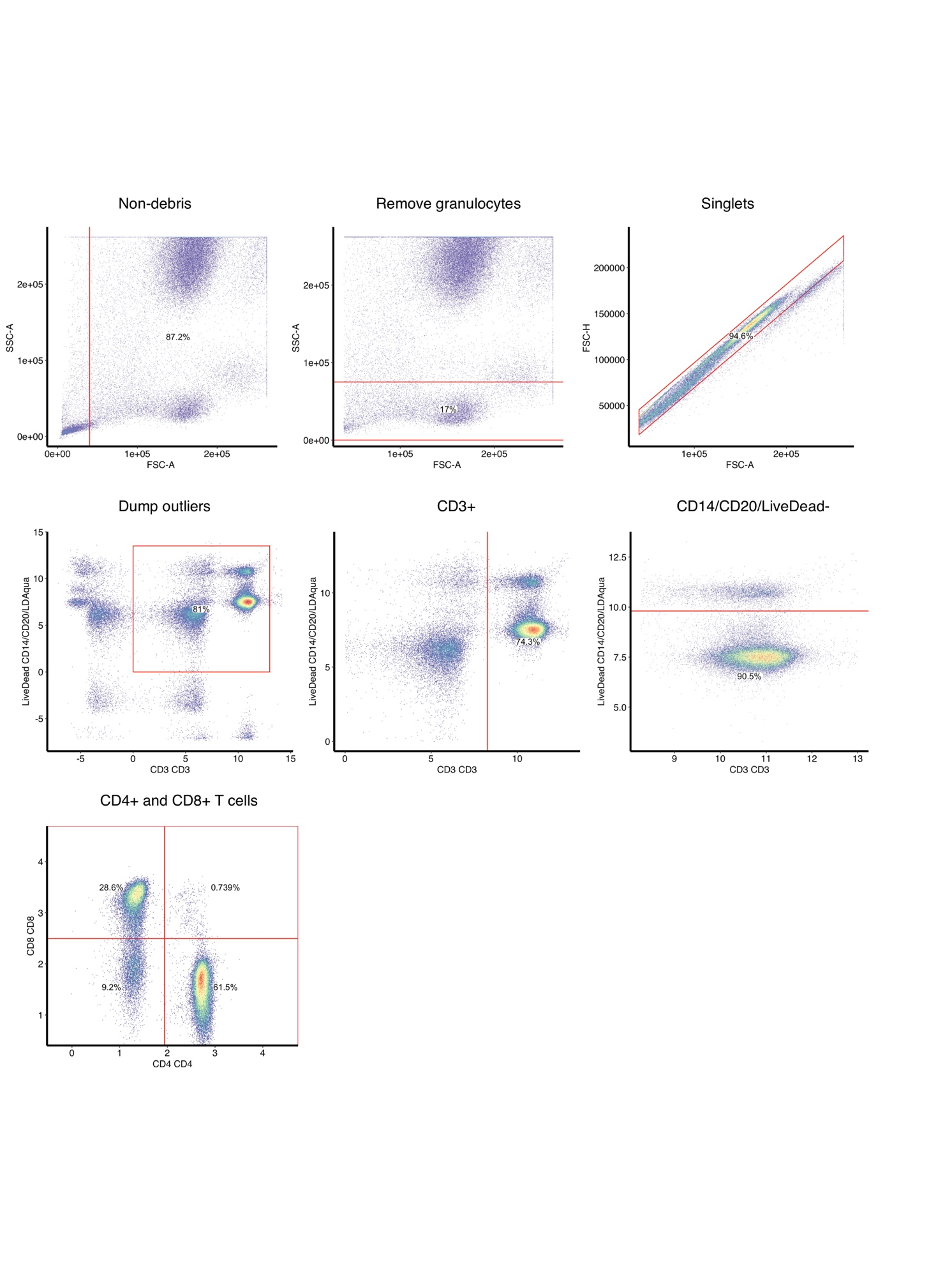
Supplemental Figure 2**

**Supplemental Figure 2. Flow cytometry plots showing auto-gating strategy using R openCyto package to extract CD4+ T cells.** Non-debris were filtered by a mindensity gate using forward and side scatter properties. Granulocytes were removed by a boundary gate. Cells were then gated for singlets with FSC-A and FSC-H by a singletGate. A clean-up boundary gate was then applied on CD3 and CD14/CD20/LiveDead parameters for better down-stream gating. CD3+ and CD14-CD20- live cells were then sequentially selected with mindensity gates. CD4+ and CD8+ T cells were discriminated based on the CD4 versus CD8 quadrant gate.

**Supplemental Figure 3**


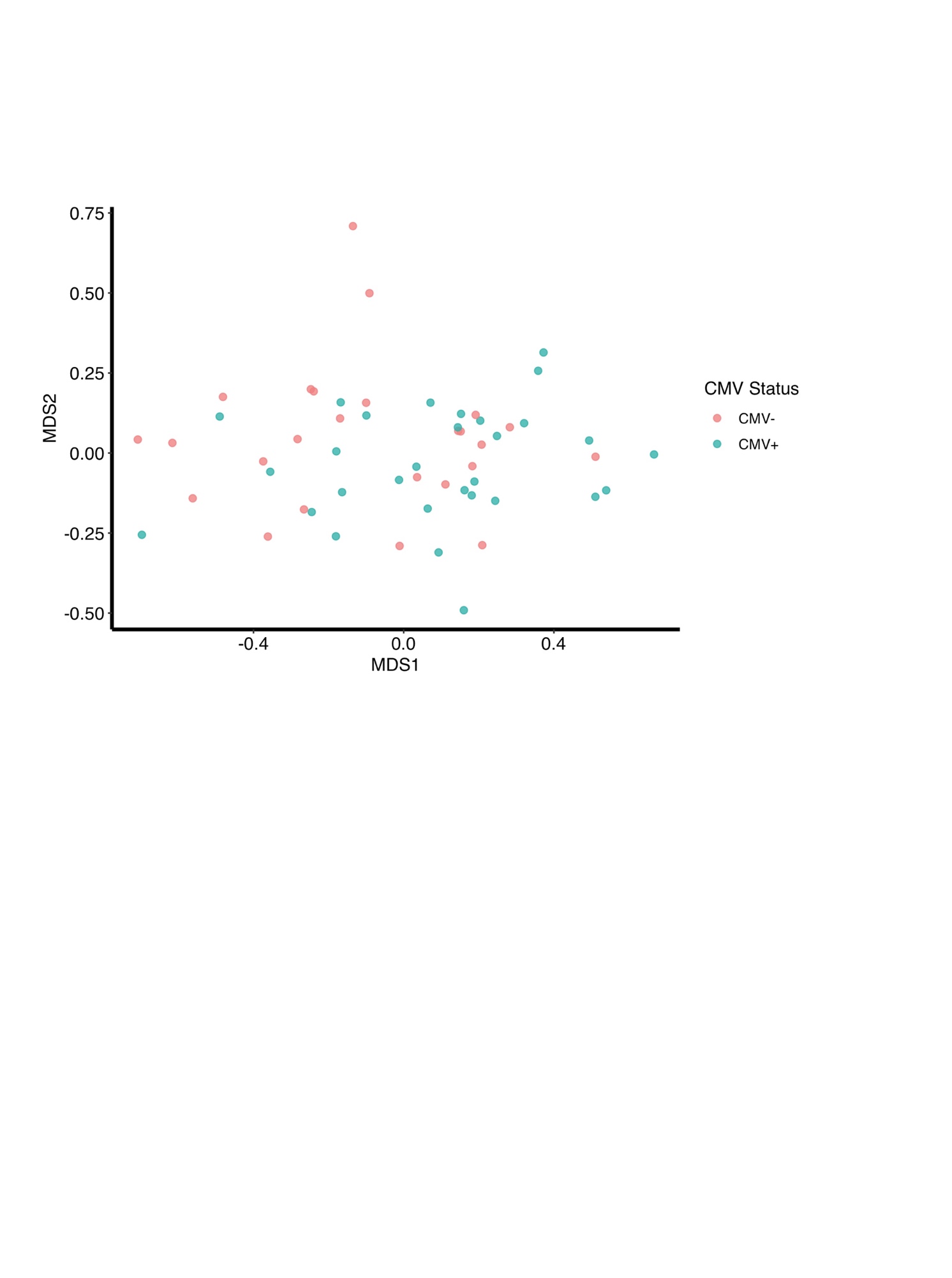


**Supplemental Figure 3: MDS plot for the 30 CMV+ and 25 CMV- samples to detect subjects with abnormal cell surface marker expression pattern.** No outlier subjects were noticed. The two-dimensional distances between samples in this plot are calculated based on the median marker expression level of 11 cell surface markers across all cells measured for each sample.

**Supplemental Figure 4**


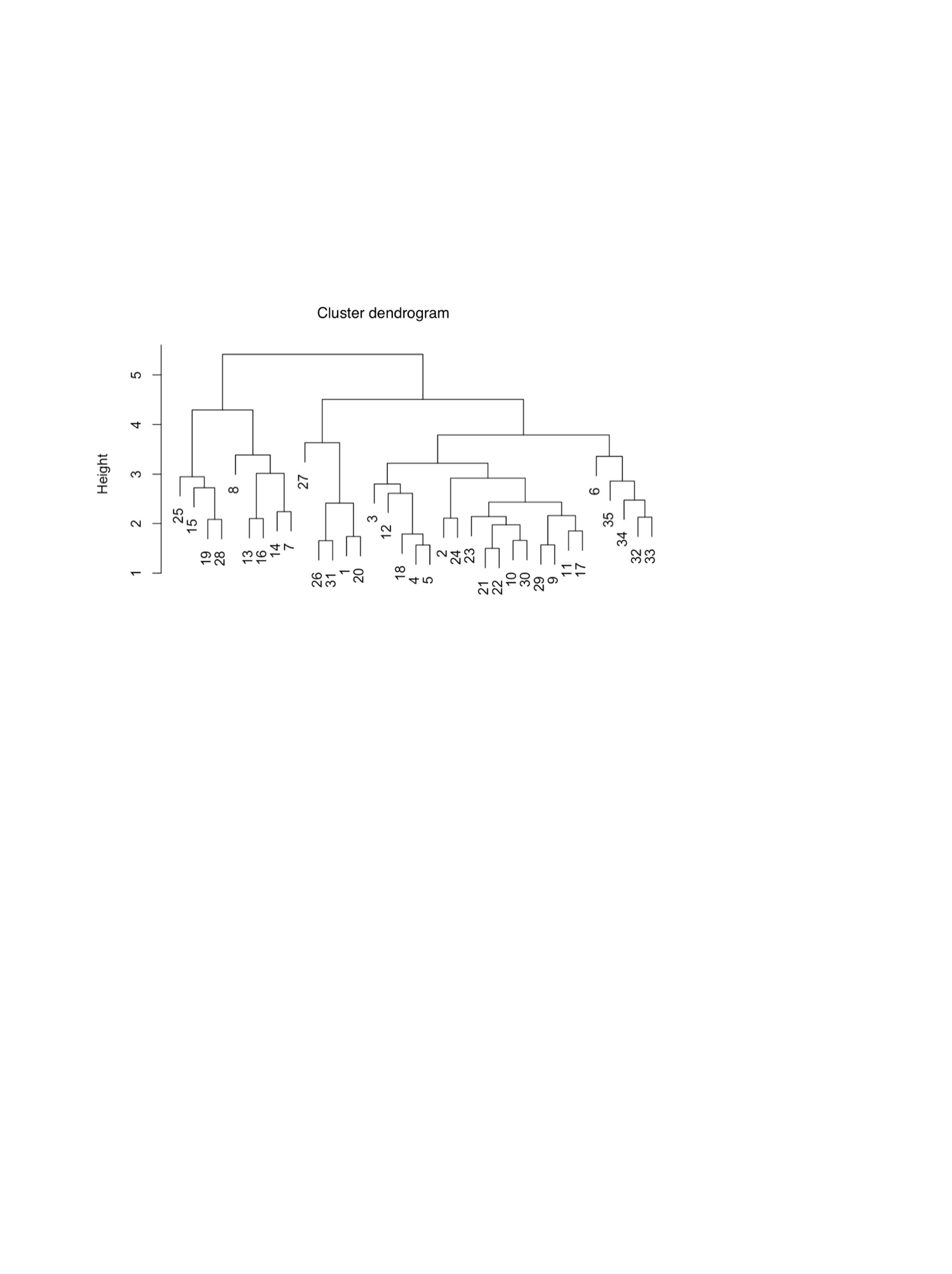


**Supplemental Figure 4: Dendrogram plot for the initially identified 35 clusters by FlowSOM algorithm.** Initially 35 clusters were identified using R FlowSOM package. These clusters are then re-grouped to 16 clusters based on similarities in the expression patterns of 11 cell surface markers.

**Supplemental Figure 5**


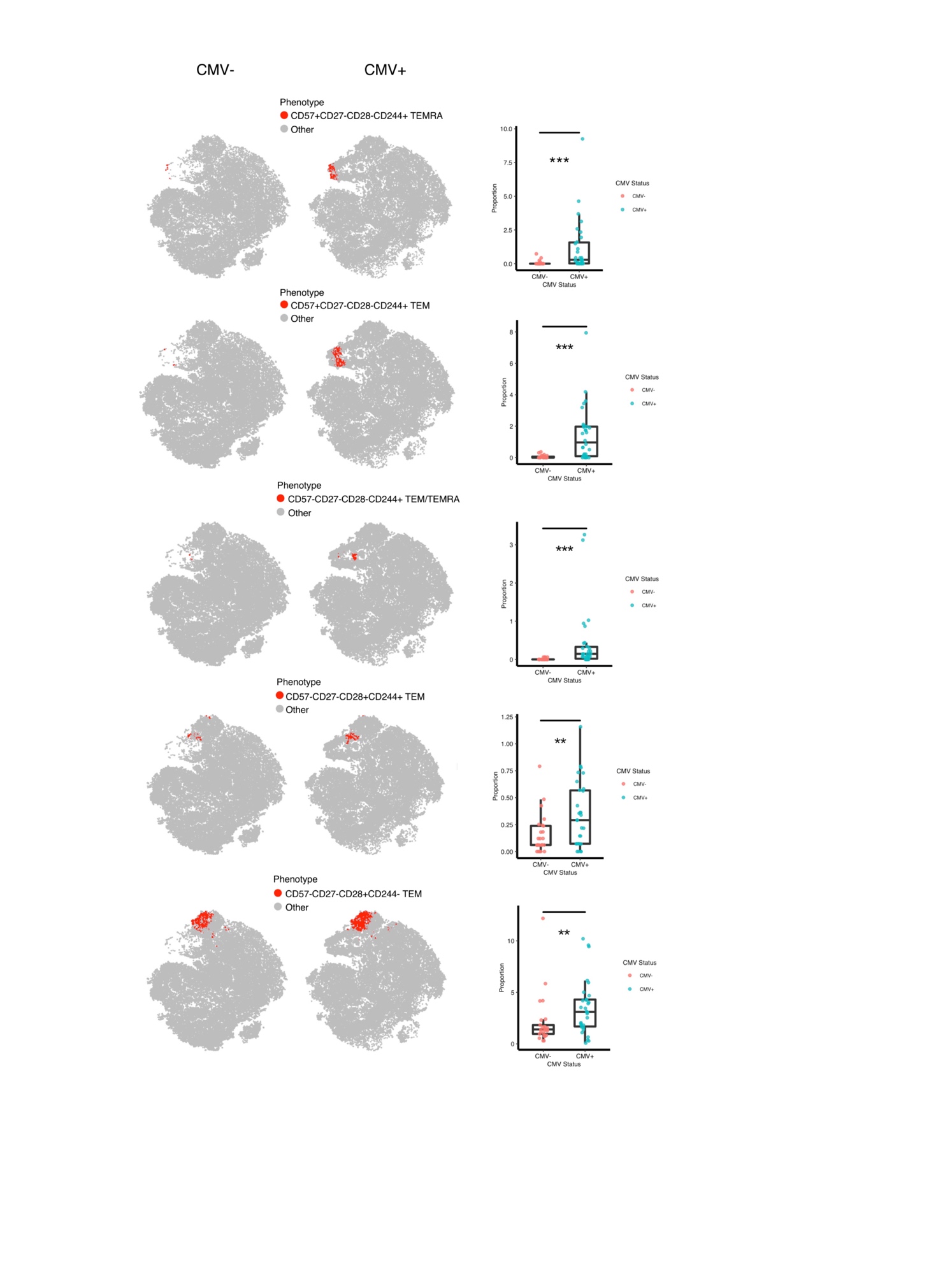


**Supplemental Figure 5: Conventional bimodal gating analysis is consistent with high dimensional flow cytometry analysis.** First column: same tSNE projection as in Figure 3(A), with bimodally gated CD57+CD27-CD28-CD244+ TEMRA, CD57+CD27-CD28-CD244+ TEM, CD57-CD27-CD28-CD244+ TEM/TEMRA, CD57-CD27-CD28+CD244+ TEM, CD57-CD27-CD28+CD244- TEM colored in red and stratified by CMV status. Second column: Statistic comparison of frequency of the corresponding subpopulations between CMV+ and CMV- healthy subjects, represented by a summary box and dot plot.

**Supplemental Figure 6**


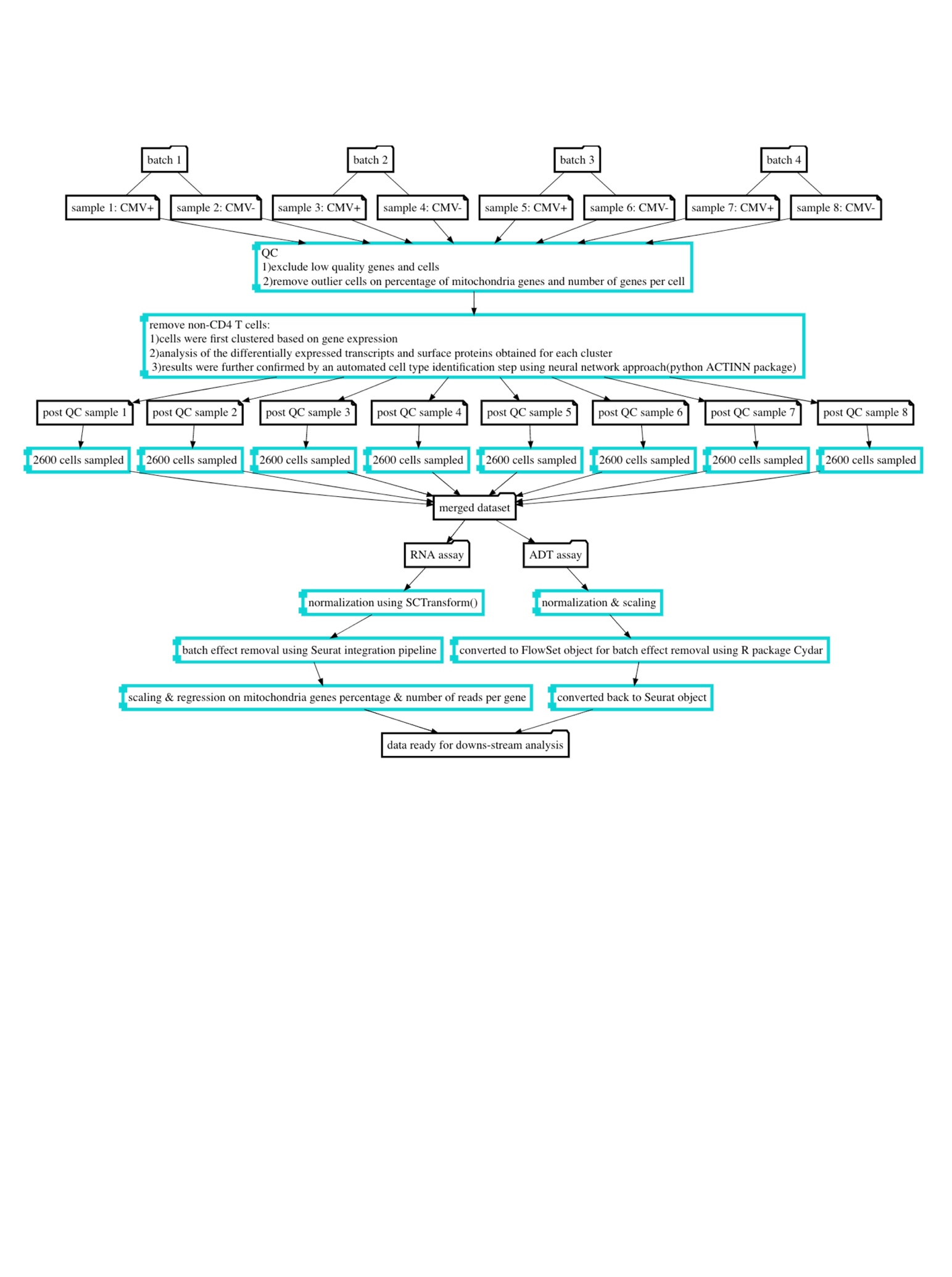


**Supplemental Figure 6: Workflow graphs showing pre-processing steps for single-cell RNA sequencing RNA and ADT data.**


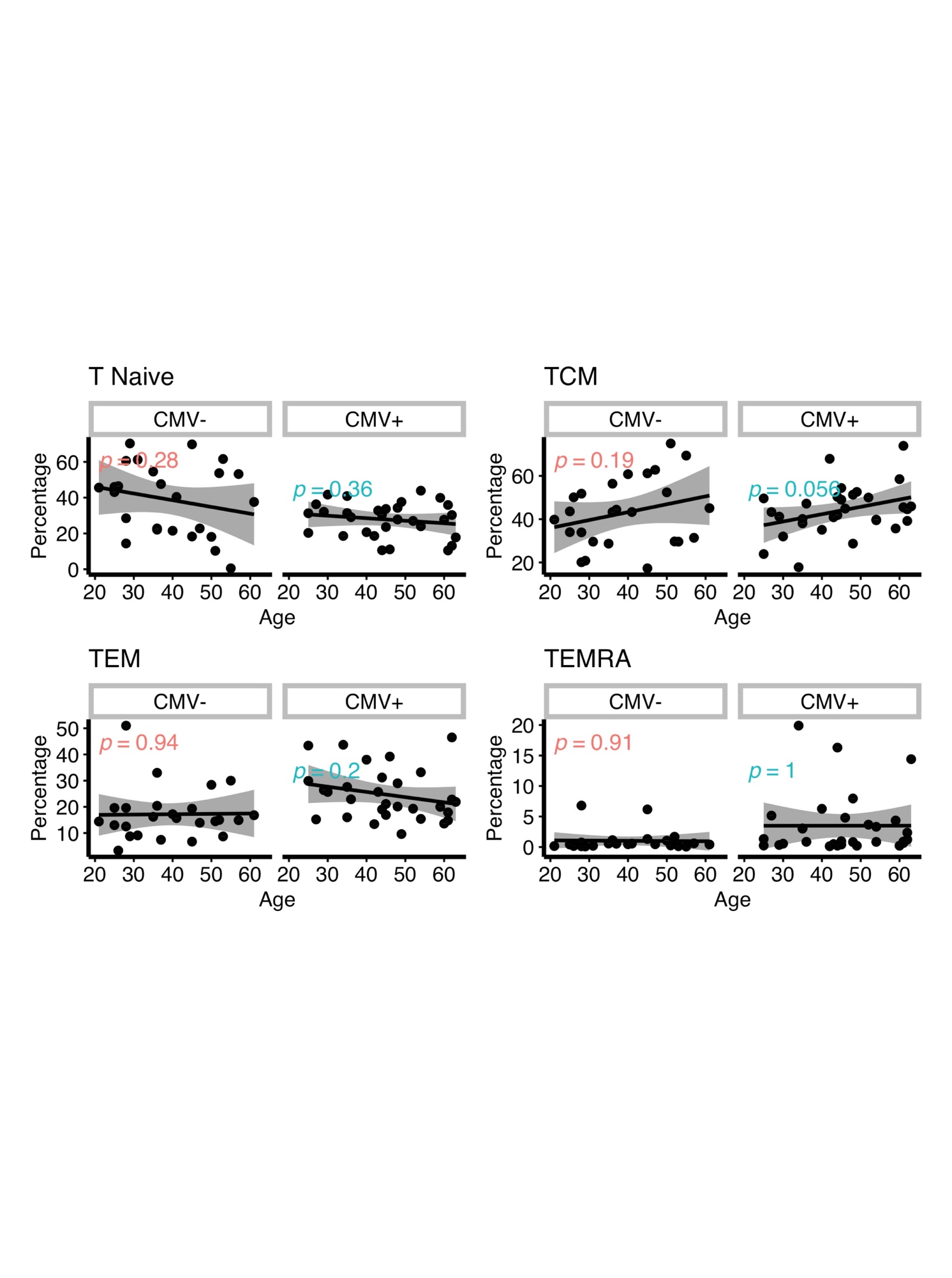
**Supplemental Figure 7**

**Supplemental Figure 7: Age does not play a significant role in memory expansion and naive cell contraction observed in CMV+ healthy subjects.** Pearson correlation test between age and percentage of memory subsets (Naive T cells, TCM, TEM and TEMRA) in CMV+ and CMV- healthy subjects, represented by dot and line plots.

**Supplemental Figure 8**

**
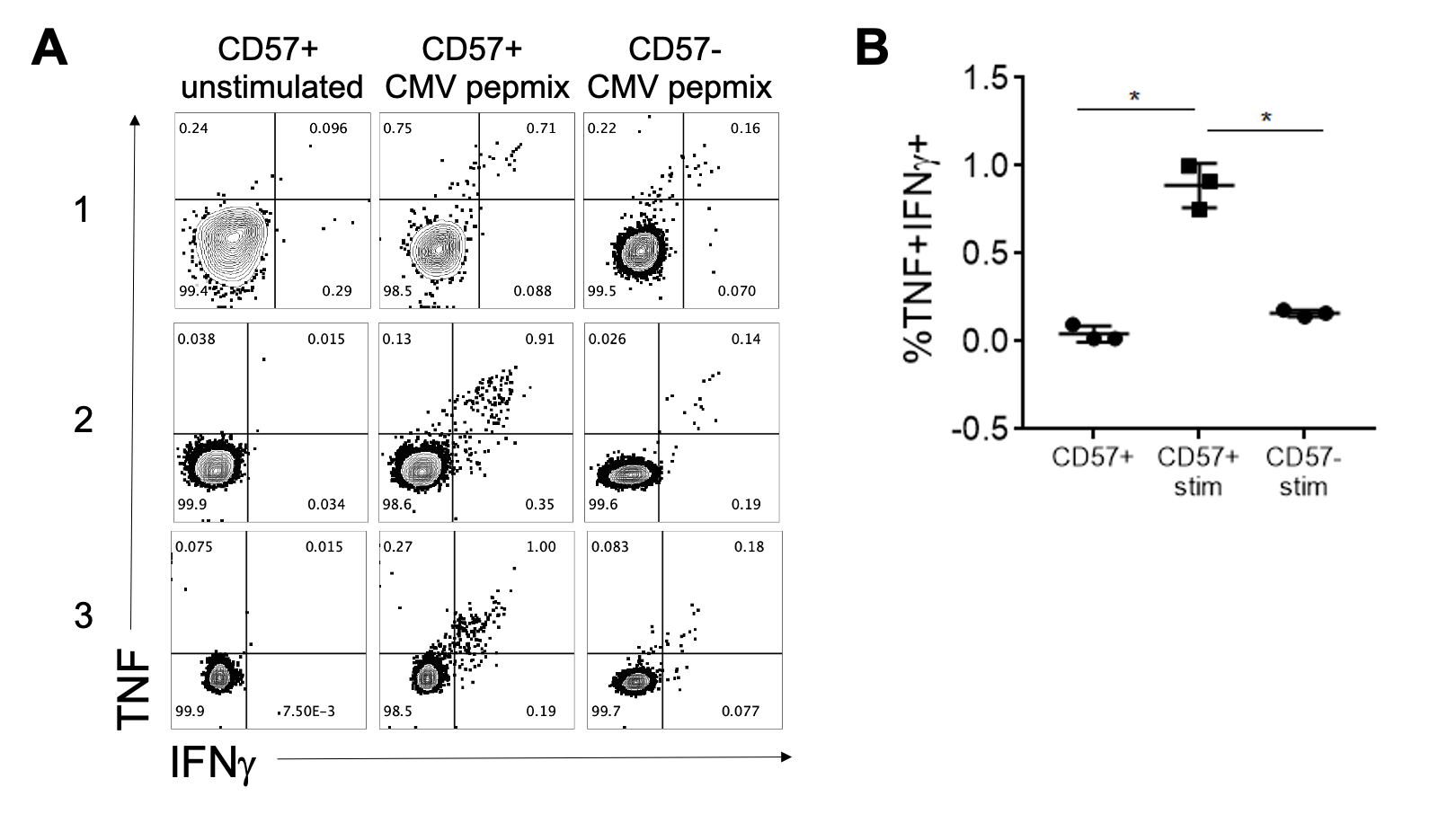
**

**Supplemental Figure 8: CMV-seropositive individuals harbor CD57+ CD4+ T cells that respond to CMV peptides.** PBMCs from CMV+ individuals were stimulated with pp65 and IEL PepMixes for 8 hours in the presence of GolgiStop and assessed for cytokine production via flow cytometry. (A) Flow cytometry plots of TNF and IFNγ cytokine production from CD4+CD57+ unstimulated T cells, CD4+CD57+ peptide-stimulated T cells, and CD4+CD57- peptide-stimulated T cells of three CMV-seropositive healthy individuals. (B) Summary data of the frequency of TNF+IFNγ+ cytokine producers of the CD4+CD57+ unstimulated T cells, CD4+CD57+ peptide-stimulated T cells, and CD4+CD57- peptide-stimulated T cells. Repeated measures one-way ANOVA with Holm's Sidak multiple comparisons was performed in GraphPad Prism version 7 (n=3, *p<0.05).

**Supplemental Figure 9**

**Supplemental Figure 9: Longitudinal analysis of memory CD4+CD57+ T cells.** (A) Changes of the frequencies of CD4+CD57+ TEM and CD4+CD57+ TEMRA subsets over time in stable, CMV+ transplant recipients on belatacept. Samples were collected at 2 months interval. (B) Changes of the frequencies of CD4+CD57+ TEM and CD4+CD57+ TEMRA subsets over time in CMV+ healthy subjects. Samples were collected at 6 months interval. (C) Statistical comparison of frequencies of the CD4+CD57+ TEM and CD4+CD57+ TEMRA subsets between CMV+ healthy subjects and stable, CMV+ transplant recipients on belatacept at follow-up time point 0, represented by a summary box and dot plot.

## Supplementary Tables

**Supplemental Table 1**

| gene | p_val | avg_logFC | pct.1 | pct.2 | p_val_adj | cluster |
| --- | --- | --- | --- | --- | --- | --- |
| CCR7 | 0 | 0.49836126 | 0.733 | 0.302 | 0 | TN/TCM |
| SELL | 0 | 0.4316257 | 0.766 | 0.37 | 0 | TN/TCM |
| LEF1 | 0 | 0.41182801 | 0.664 | 0.31 | 0 | TN/TCM |
| TCF7 | 0 | 0.3831309 | 0.683 | 0.355 | 0 | TN/TCM |
| CD7 | 0 | 0.37579958 | 0.661 | 0.349 | 0 | TN/TCM |
| RPS13 | 0 | 0.35780694 | 0.858 | 0.281 | 0 | TN/TCM |
| EEF1B2 | 0 | 0.31895538 | 0.755 | 0.36 | 0 | TN/TCM |
| FHIT | 0 | 0.31444573 | 0.513 | 0.156 | 0 | TN/TCM |
| RPS3A | 0 | 0.3097799 | 0.853 | 0.284 | 0 | TN/TCM |
| RPS5 | 0 | 0.30871878 | 0.789 | 0.334 | 0 | TN/TCM |
| PIK3IP1 | 0 | 0.30297653 | 0.678 | 0.436 | 0 | TN/TCM |
| PABPC1 | 0 | 0.29097219 | 0.752 | 0.39 | 0 | TN/TCM |
| NDFIP1 | 0 | 0.28322089 | 0.606 | 0.361 | 0 | TN/TCM |
| SARAF | 0 | 0.2831737 | 0.715 | 0.39 | 0 | TN/TCM |
| ADTRP | 0 | 0.26826721 | 0.463 | 0.103 | 0 | TN/TCM |
| RPL32 | 0 | 0.26463732 | 0.85 | 0.32 | 0 | TN/TCM |
| RPL5 | 0 | 0.25665692 | 0.757 | 0.352 | 0 | TN/TCM |
| RPS23 | 0 | 0.25353903 | 0.798 | 0.299 | 0 | TN/TCM |
| ABLIM1 | 4.97E-288 | 0.25031288 | 0.528 | 0.318 | 1.49E-284 | TN/TCM |
| FOS | 0 | 0.7813897 | 0.699 | 0.233 | 0 | TEM |
| S100A4 | 0 | 0.63742087 | 0.778 | 0.295 | 0 | TEM |
| GZMK | 0 | 0.58045859 | 0.262 | 0.038 | 0 | TEM |
| DUSP1 | 0 | 0.56854849 | 0.687 | 0.277 | 0 | TEM |
| ANXA1 | 0 | 0.55601349 | 0.722 | 0.28 | 0 | TEM |
| S100A11 | 0 | 0.529314 | 0.776 | 0.361 | 0 | TEM |
| IL32 | 0 | 0.52513886 | 0.772 | 0.303 | 0 | TEM |
| ZFP36 | 0 | 0.51053009 | 0.676 | 0.355 | 0 | TEM |
| AHNAK | 0 | 0.47125677 | 0.703 | 0.273 | 0 | TEM |
| ITGB1 | 0 | 0.46890516 | 0.649 | 0.254 | 0 | TEM |
| IL7R | 0 | 0.46180708 | 0.703 | 0.387 | 0 | TEM |
| KLF6 | 0 | 0.42750262 | 0.696 | 0.349 | 0 | TEM |
| DUSP2 | 0 | 0.41194352 | 0.586 | 0.255 | 0 | TEM |
| COTL1 | 0 | 0.40220831 | 0.672 | 0.339 | 0 | TEM |
| S100A10 | 0 | 0.37003722 | 0.721 | 0.386 | 0 | TEM |
| JUN | 0 | 0.36128002 | 0.594 | 0.362 | 0 | TEM |
| TNFAIP3 | 0 | 0.35878183 | 0.586 | 0.274 | 0 | TEM |
| SH3BGRL3 | 0 | 0.35177653 | 0.778 | 0.344 | 0 | TEM |
| CD69 | 0 | 0.34067188 | 0.595 | 0.316 | 0 | TEM |
| PPP1R15A | 0 | 0.32595435 | 0.59 | 0.204 | 0 | TEM |
| ANXA2 | 0 | 0.32386675 | 0.546 | 0.167 | 0 | TEM |
| VIM | 0 | 0.32094658 | 0.67 | 0.434 | 0 | TEM |
| TAGLN2 | 0 | 0.32012745 | 0.686 | 0.414 | 0 | TEM |
| CLIC1 | 0 | 0.30077712 | 0.703 | 0.406 | 0 | TEM |
| S100A6 | 0 | 0.2827745 | 0.685 | 0.433 | 0 | TEM |
| EMP3 | 0 | 0.27601538 | 0.703 | 0.432 | 0 | TEM |
| IL10RA | 0 | 0.27009668 | 0.613 | 0.332 | 0 | TEM |
| LYAR | 0 | 0.263641 | 0.407 | 0.171 | 0 | TEM |
| CD99 | 0 | 0.25307477 | 0.652 | 0.362 | 0 | TEM |
| TRADD | 1.85E-292 | 0.29141932 | 0.578 | 0.267 | 5.54E-289 | TEM |
| KLRB1 | 7.53E-292 | 0.66246208 | 0.45 | 0.086 | 2.26E-288 | TEM |
| TIMP1 | 1.61E-290 | 0.30295744 | 0.521 | 0.248 | 4.83E-287 | TEM |
| PLP2 | 9.14E-275 | 0.2899603 | 0.625 | 0.386 | 2.74E-271 | TEM |
| ZFP36L2 | 1.01E-263 | 0.30067442 | 0.655 | 0.457 | 3.02E-260 | TEM |
| ARHGAP15 | 1.35E-252 | 0.25736107 | 0.639 | 0.422 | 4.06E-249 | TEM |
| GSTK1 | 9.08E-249 | 0.25629736 | 0.647 | 0.442 | 2.72E-245 | TEM |
| CXCR4 | 7.45E-229 | 0.2889561 | 0.571 | 0.398 | 2.23E-225 | TEM |
| LIMS1 | 2.61E-193 | 0.25478175 | 0.456 | 0.199 | 7.82E-190 | TEM |
| AQP3 | 1.45E-192 | 0.28445292 | 0.616 | 0.416 | 4.36E-189 | TEM |
| LTB | 2.00E-171 | 0.25947143 | 0.652 | 0.512 | 5.99E-168 | TEM |
| NKG7 | 0 | 2.92885283 | 1 | 0.085 | 0 | Tdiff |
| GNLY | 0 | 2.62052115 | 0.935 | 0.048 | 0 | Tdiff |
| CCL5 | 0 | 2.22622864 | 1 | 0.145 | 0 | Tdiff |
| GZMH | 0 | 2.08994013 | 0.99 | 0.045 | 0 | Tdiff |
| FGFBP2 | 0 | 1.72175642 | 0.961 | 0.033 | 0 | Tdiff |
| CST7 | 0 | 1.59774446 | 0.988 | 0.147 | 0 | Tdiff |
| GZMA | 0 | 1.47447798 | 0.988 | 0.152 | 0 | Tdiff |
| PRF1 | 0 | 1.31632754 | 0.931 | 0.129 | 0 | Tdiff |
| CCL4 | 0 | 0.95509847 | 0.81 | 0.044 | 0 | Tdiff |
| S100A41 | 0 | 0.95476971 | 0.992 | 0.466 | 0 | Tdiff |
| SH3BGRL31 | 0 | 0.94695845 | 0.992 | 0.496 | 0 | Tdiff |
| GZMB | 0 | 0.91067991 | 0.738 | 0.025 | 0 | Tdiff |
| IL321 | 0 | 0.87351675 | 0.99 | 0.468 | 0 | Tdiff |
| HOPX | 0 | 0.86247669 | 0.843 | 0.128 | 0 | Tdiff |
| PLEK | 0 | 0.81815133 | 0.803 | 0.067 | 0 | Tdiff |
| SRGN | 0 | 0.78850949 | 0.934 | 0.505 | 0 | Tdiff |
| HCST | 0 | 0.7344727 | 0.898 | 0.491 | 0 | Tdiff |
| CD991 | 0 | 0.73063908 | 0.928 | 0.454 | 0 | Tdiff |
| HLA-C | 0 | 0.72441945 | 0.985 | 0.487 | 0 | Tdiff |
| ITGB2 | 0 | 0.68891496 | 0.924 | 0.53 | 0 | Tdiff |
| HLA-DPB1 | 0 | 0.68242481 | 0.811 | 0.208 | 0 | Tdiff |
| HLA-A | 0 | 0.67339964 | 0.984 | 0.469 | 0 | Tdiff |
| ZNF683 | 0 | 0.66298858 | 0.6 | 0.037 | 0 | Tdiff |
| ADGRG1 | 0 | 0.6565158 | 0.721 | 0.029 | 0 | Tdiff |
| SLC9A3R1 | 0 | 0.64190047 | 0.893 | 0.511 | 0 | Tdiff |
| CX3CR1 | 0 | 0.61965514 | 0.682 | 0.034 | 0 | Tdiff |
| CYBA | 0 | 0.60488379 | 0.903 | 0.514 | 0 | Tdiff |
| ZEB2 | 0 | 0.59693907 | 0.676 | 0.048 | 0 | Tdiff |
| S1PR5 | 0 | 0.59585204 | 0.715 | 0.038 | 0 | Tdiff |
| B2M | 0 | 0.59552491 | 0.999 | 0.434 | 0 | Tdiff |
| PFN1 | 0 | 0.5609776 | 0.933 | 0.493 | 0 | Tdiff |
| CD52 | 0 | 0.54412034 | 0.933 | 0.488 | 0 | Tdiff |
| PRSS23 | 0 | 0.53158024 | 0.665 | 0.021 | 0 | Tdiff |
| FCRL6 | 0 | 0.52560277 | 0.699 | 0.069 | 0 | Tdiff |
| HLA-B | 0 | 0.51355777 | 0.968 | 0.479 | 0 | Tdiff |
| C1orf21 | 0 | 0.48015358 | 0.664 | 0.044 | 0 | Tdiff |
| MT-CO1 | 0 | 0.42844955 | 0.942 | 0.478 | 0 | Tdiff |
| FGR | 0 | 0.37190628 | 0.587 | 0.045 | 0 | Tdiff |
| TMSB4X | 0 | 0.33083474 | 0.916 | 0.483 | 0 | Tdiff |
| GZMM | 1.57E-299 | 0.63668205 | 0.827 | 0.355 | 4.70E-296 | Tdiff |
| LGALS1 | 9.56E-295 | 0.85510013 | 0.811 | 0.233 | 2.87E-291 | Tdiff |
| C12orf75 | 4.15E-292 | 0.64958482 | 0.79 | 0.226 | 1.25E-288 | Tdiff |
| EFHD2 | 2.84E-291 | 0.60372488 | 0.732 | 0.131 | 8.51E-288 | Tdiff |
| MATK | 1.49E-282 | 0.585158 | 0.746 | 0.137 | 4.48E-279 | Tdiff |
| SPON2 | 2.27E-276 | 0.7513883 | 0.712 | 0.073 | 6.81E-273 | Tdiff |
| KLRG1 | 6.48E-274 | 0.65245287 | 0.791 | 0.222 | 1.94E-270 | Tdiff |
| IFITM3 | 4.65E-263 | 0.61367045 | 0.789 | 0.386 | 1.40E-259 | Tdiff |
| S100A101 | 4.64E-260 | 0.54609711 | 0.907 | 0.502 | 1.39E-256 | Tdiff |
| LITAF | 4.92E-255 | 0.58878791 | 0.875 | 0.524 | 1.48E-251 | Tdiff |
| CLIC11 | 5.28E-246 | 0.59562167 | 0.89 | 0.508 | 1.58E-242 | Tdiff |
| CTSW | 2.40E-245 | 0.66899351 | 0.774 | 0.264 | 7.20E-242 | Tdiff |
| HLA-DPA1 | 1.88E-243 | 0.54948597 | 0.728 | 0.192 | 5.65E-240 | Tdiff |
| FLNA | 3.84E-241 | 0.61468424 | 0.833 | 0.425 | 1.15E-237 | Tdiff |
| ANXA11 | 3.75E-235 | 0.58589977 | 0.839 | 0.443 | 1.13E-231 | Tdiff |
| ASCL2 | 5.22E-233 | 0.35471718 | 0.539 | 0.049 | 1.56E-229 | Tdiff |
| LINC01871 | 1.93E-226 | 0.50591515 | 0.713 | 0.162 | 5.79E-223 | Tdiff |
| TBX21 | 6.34E-224 | 0.54108687 | 0.674 | 0.098 | 1.90E-220 | Tdiff |
| EMP31 | 4.78E-221 | 0.47410877 | 0.872 | 0.525 | 1.43E-217 | Tdiff |
| LYAR1 | 1.18E-219 | 0.56555155 | 0.74 | 0.238 | 3.53E-216 | Tdiff |
| MYL6 | 1.75E-218 | 0.42589077 | 0.86 | 0.518 | 5.26E-215 | Tdiff |
| S100A61 | 1.99E-213 | 0.44986574 | 0.862 | 0.518 | 5.98E-210 | Tdiff |
| CFL1 | 8.27E-208 | 0.40106714 | 0.827 | 0.512 | 2.48E-204 | Tdiff |
| RPS26 | 1.14E-205 | 0.29613139 | 0.865 | 0.496 | 3.41E-202 | Tdiff |
| MT2A | 6.34E-205 | 0.63666644 | 0.818 | 0.41 | 1.90E-201 | Tdiff |
| MT-ATP6 | 7.16E-205 | 0.31959037 | 0.831 | 0.499 | 2.15E-201 | Tdiff |
| LSP1 | 1.39E-195 | 0.43214087 | 0.832 | 0.514 | 4.16E-192 | Tdiff |
| CALM1 | 3.33E-194 | 0.3748357 | 0.846 | 0.526 | 9.98E-191 | Tdiff |
| AHNAK1 | 6.43E-193 | 0.56902564 | 0.822 | 0.431 | 1.93E-189 | Tdiff |
| YWHAQ | 1.59E-186 | 0.48707872 | 0.779 | 0.421 | 4.77E-183 | Tdiff |
| ACTB | 1.71E-183 | 0.4698232 | 0.822 | 0.505 | 5.12E-180 | Tdiff |
| APOBEC3G | 3.45E-181 | 0.49296808 | 0.68 | 0.201 | 1.03E-177 | Tdiff |
| TGFBR3 | 6.24E-175 | 0.46233889 | 0.629 | 0.126 | 1.87E-171 | Tdiff |
| ITGB11 | 1.79E-173 | 0.50346936 | 0.807 | 0.395 | 5.36E-170 | Tdiff |
| CTSC | 2.52E-172 | 0.47670148 | 0.707 | 0.301 | 7.56E-169 | Tdiff |
| TPST2 | 6.91E-172 | 0.37622178 | 0.622 | 0.168 | 2.07E-168 | Tdiff |
| ARPC2 | 2.90E-170 | 0.39727656 | 0.835 | 0.523 | 8.71E-167 | Tdiff |
| PTPRC | 2.59E-160 | 0.3636334 | 0.798 | 0.514 | 7.76E-157 | Tdiff |
| ADRB2 | 2.08E-151 | 0.34068454 | 0.539 | 0.078 | 6.24E-148 | Tdiff |
| HLA-E | 3.88E-148 | 0.28460194 | 0.823 | 0.533 | 1.16E-144 | Tdiff |
| SAMD3 | 1.19E-137 | 0.42630329 | 0.623 | 0.183 | 3.56E-134 | Tdiff |
| MT-ND5 | 1.04E-136 | 0.30015802 | 0.78 | 0.51 | 3.12E-133 | Tdiff |
| RARRES3 | 7.28E-136 | 0.39021529 | 0.732 | 0.48 | 2.18E-132 | Tdiff |
| UBC | 1.00E-134 | 0.33032748 | 0.799 | 0.535 | 3.00E-131 | Tdiff |
| TRBV3-1 | 9.25E-134 | 1.06023637 | 0.406 | 0.052 | 2.78E-130 | Tdiff |
| CD300A | 4.07E-132 | 0.38803945 | 0.6 | 0.147 | 1.22E-128 | Tdiff |
| CD74 | 2.17E-131 | 0.49787322 | 0.741 | 0.464 | 6.52E-128 | Tdiff |
| AOAH | 3.85E-131 | 0.30292238 | 0.539 | 0.083 | 1.16E-127 | Tdiff |
| CD2 | 1.03E-125 | 0.3783084 | 0.714 | 0.471 | 3.10E-122 | Tdiff |
| BHLHE40 | 6.03E-125 | 0.3730682 | 0.679 | 0.244 | 1.81E-121 | Tdiff |
| CD320 | 9.09E-125 | 0.38208648 | 0.634 | 0.245 | 2.73E-121 | Tdiff |
| MYO1G | 6.75E-121 | 0.36587039 | 0.657 | 0.318 | 2.02E-117 | Tdiff |
| SPN | 9.37E-121 | 0.35366945 | 0.628 | 0.344 | 2.81E-117 | Tdiff |
| LCP1 | 3.99E-115 | 0.406684 | 0.81 | 0.545 | 1.20E-111 | Tdiff |
| LAIR2 | 1.21E-112 | 0.3891549 | 0.57 | 0.107 | 3.63E-109 | Tdiff |
| PPP1CA | 1.96E-111 | 0.35513231 | 0.78 | 0.532 | 5.88E-108 | Tdiff |
| PAXX | 3.57E-111 | 0.37424591 | 0.712 | 0.395 | 1.07E-107 | Tdiff |
| UBB | 1.25E-107 | 0.33941534 | 0.733 | 0.496 | 3.76E-104 | Tdiff |
| MT-ND4 | 1.09E-106 | 0.29539722 | 0.728 | 0.508 | 3.26E-103 | Tdiff |
| LY6E | 5.90E-106 | 0.37235323 | 0.8 | 0.54 | 1.77E-102 | Tdiff |
| MSN | 5.44E-102 | 0.36181043 | 0.741 | 0.491 | 1.63E-98 | Tdiff |
| APMAP | 1.75E-99 | 0.31438914 | 0.562 | 0.214 | 5.24E-96 | Tdiff |
| CHST12 | 1.42E-95 | 0.32635946 | 0.522 | 0.175 | 4.27E-92 | Tdiff |
| CLEC2B | 7.32E-95 | 0.34267217 | 0.678 | 0.353 | 2.20E-91 | Tdiff |
| ID2 | 8.75E-94 | 0.3984189 | 0.615 | 0.258 | 2.63E-90 | Tdiff |
| ANXA21 | 1.30E-93 | 0.35304072 | 0.662 | 0.305 | 3.91E-90 | Tdiff |
| S100A111 | 7.39E-93 | 0.31554238 | 0.821 | 0.518 | 2.22E-89 | Tdiff |
| PSME1 | 6.25E-88 | 0.25766981 | 0.714 | 0.508 | 1.88E-84 | Tdiff |
| TGFB1 | 1.02E-87 | 0.30573764 | 0.644 | 0.351 | 3.05E-84 | Tdiff |
| SYNE1 | 7.26E-87 | 0.29695084 | 0.552 | 0.236 | 2.18E-83 | Tdiff |
| PPP2R5C | 1.96E-84 | 0.30386411 | 0.669 | 0.4 | 5.89E-81 | Tdiff |
| PSMB9 | 2.16E-83 | 0.29711037 | 0.774 | 0.548 | 6.48E-80 | Tdiff |
| MYO1F | 9.77E-82 | 0.35669135 | 0.555 | 0.184 | 2.93E-78 | Tdiff |
| IFITM2 | 2.64E-78 | 0.31636987 | 0.736 | 0.525 | 7.93E-75 | Tdiff |
| PTGDR | 1.52E-75 | 0.25418778 | 0.49 | 0.087 | 4.55E-72 | Tdiff |
| ITGAM | 1.00E-73 | 0.25686325 | 0.459 | 0.086 | 3.01E-70 | Tdiff |
| PPP1R18 | 4.38E-71 | 0.27826719 | 0.665 | 0.461 | 1.31E-67 | Tdiff |
| PPIB | 1.28E-70 | 0.25442455 | 0.709 | 0.502 | 3.83E-67 | Tdiff |
| TAGLN21 | 1.99E-70 | 0.2912716 | 0.741 | 0.515 | 5.98E-67 | Tdiff |
| ITGAL | 1.56E-67 | 0.29079477 | 0.534 | 0.233 | 4.67E-64 | Tdiff |
| SH2D2A | 2.56E-67 | 0.28955168 | 0.526 | 0.213 | 7.69E-64 | Tdiff |
| ANXA6 | 3.83E-66 | 0.27791448 | 0.756 | 0.548 | 1.15E-62 | Tdiff |
| ACTN4 | 1.65E-65 | 0.28153688 | 0.503 | 0.193 | 4.96E-62 | Tdiff |
| STOM | 2.09E-65 | 0.28646131 | 0.515 | 0.189 | 6.26E-62 | Tdiff |
| CAST | 9.35E-64 | 0.26771642 | 0.622 | 0.395 | 2.80E-60 | Tdiff |
| CYTOR | 3.29E-63 | 0.29837806 | 0.532 | 0.154 | 9.88E-60 | Tdiff |
| PYHIN1 | 4.66E-59 | 0.2634228 | 0.53 | 0.215 | 1.40E-55 | Tdiff |
| SYNE2 | 1.15E-56 | 0.28787422 | 0.639 | 0.384 | 3.44E-53 | Tdiff |
| GSTP1 | 4.36E-55 | 0.25786563 | 0.627 | 0.396 | 1.31E-51 | Tdiff |
| GNG2 | 5.99E-55 | 0.3046678 | 0.598 | 0.31 | 1.80E-51 | Tdiff |
| ARL4C | 3.81E-54 | 0.27180993 | 0.711 | 0.492 | 1.14E-50 | Tdiff |
| RASSF1 | 2.34E-51 | 0.27847087 | 0.502 | 0.214 | 7.03E-48 | Tdiff |
| MBP | 2.36E-49 | 0.25768281 | 0.661 | 0.451 | 7.09E-46 | Tdiff |
| CALR | 8.54E-49 | 0.28826735 | 0.725 | 0.533 | 2.56E-45 | Tdiff |
| MYBL1 | 2.72E-45 | 0.25315278 | 0.413 | 0.11 | 8.15E-42 | Tdiff |
| TRBV14 | 1.38E-33 | 0.32409936 | 0.329 | 0.116 | 4.14E-30 | Tdiff |
| HLA-DRB1 | 6.32E-25 | 0.27425949 | 0.443 | 0.132 | 1.90E-21 | Tdiff |
| DUSP21 | 4.54E-21 | 0.29923831 | 0.611 | 0.382 | 1.36E-17 | Tdiff |
| TRAV13-1 | 1.08E-07 | 0.31874477 | 0.171 | 0.099 | 0.00032298 | Tdiff |
| TRAV23DV6 | 0.00027843 | 0.27946732 | 0.162 | 0.12 | 0.83529998 | Tdiff |
| TRAV12-1 | 0.00060109 | 0.39027104 | 0.202 | 0.093 | 1 | Tdiff |
| RGS1 | 3.00E-178 | 0.35168745 | 0.722 | 0.255 | 8.99E-175 | Treg |
| FOXP3 | 1.45E-169 | 0.72801664 | 0.689 | 0.046 | 4.36E-166 | Treg |
| CTLA4 | 1.68E-129 | 0.36404885 | 0.615 | 0.141 | 5.03E-126 | Treg |
| TNFRSF1B | 2.33E-115 | 0.46456249 | 0.671 | 0.259 | 6.99E-112 | Treg |
| TIGIT | 2.95E-101 | 0.47421766 | 0.607 | 0.126 | 8.84E-98 | Treg |
| LGALS3 | 1.52E-96 | 0.37600821 | 0.453 | 0.121 | 4.55E-93 | Treg |
| IL10RA1 | 1.98E-90 | 0.41285419 | 0.757 | 0.437 | 5.93E-87 | Treg |
| CYTOR1 | 1.95E-87 | 0.39582863 | 0.498 | 0.164 | 5.85E-84 | Treg |
| IKZF2 | 4.19E-86 | 0.31820923 | 0.544 | 0.047 | 1.26E-82 | Treg |
| HLA-A1 | 2.47E-77 | 0.31216595 | 0.744 | 0.491 | 7.41E-74 | Treg |
| IL322 | 1.16E-76 | 0.61361642 | 0.728 | 0.492 | 3.49E-73 | Treg |
| S100A102 | 2.15E-74 | 0.39545124 | 0.759 | 0.518 | 6.45E-71 | Treg |
| RTKN2 | 3.01E-72 | 0.44700517 | 0.584 | 0.108 | 9.03E-69 | Treg |
| LSP11 | 2.61E-69 | 0.36566239 | 0.731 | 0.526 | 7.82E-66 | Treg |
| GAPDH | 7.07E-68 | 0.31170006 | 0.771 | 0.55 | 2.12E-64 | Treg |
| HLA-DRB11 | 6.26E-62 | 0.54537626 | 0.475 | 0.137 | 1.88E-58 | Treg |
| S100A42 | 1.91E-61 | 0.46077716 | 0.736 | 0.49 | 5.73E-58 | Treg |
| GBP5 | 4.44E-54 | 0.35432726 | 0.559 | 0.263 | 1.33E-50 | Treg |
| PFN11 | 2.20E-52 | 0.27716621 | 0.711 | 0.513 | 6.60E-49 | Treg |
| STAM | 3.31E-52 | 0.28111649 | 0.499 | 0.153 | 9.93E-49 | Treg |
| SHMT2 | 5.57E-52 | 0.33700497 | 0.57 | 0.222 | 1.67E-48 | Treg |
| CD741 | 4.95E-50 | 0.62015991 | 0.646 | 0.474 | 1.49E-46 | Treg |
| USP15 | 5.14E-48 | 0.32051999 | 0.604 | 0.36 | 1.54E-44 | Treg |
| MIR4435-2HG | 3.35E-47 | 0.25138681 | 0.411 | 0.108 | 1.01E-43 | Treg |
| HLA-DPA11 | 1.37E-44 | 0.34102104 | 0.473 | 0.216 | 4.11E-41 | Treg |
| RAC2 | 5.74E-43 | 0.26859419 | 0.702 | 0.539 | 1.72E-39 | Treg |
| FCMR | 7.01E-42 | 0.28386944 | 0.702 | 0.543 | 2.10E-38 | Treg |
| HLA-DQB1 | 8.68E-42 | 0.30372016 | 0.453 | 0.111 | 2.60E-38 | Treg |
| SELPLG | 5.51E-41 | 0.28116897 | 0.694 | 0.541 | 1.65E-37 | Treg |
| BIRC3 | 1.40E-40 | 0.26511689 | 0.505 | 0.281 | 4.21E-37 | Treg |
| SH3BGRL32 | 2.13E-40 | 0.26552273 | 0.696 | 0.521 | 6.40E-37 | Treg |
| HLA-DPB11 | 1.57E-38 | 0.29549148 | 0.471 | 0.237 | 4.71E-35 | Treg |
| LGALS11 | 1.42E-37 | 0.38021981 | 0.478 | 0.261 | 4.25E-34 | Treg |
| ACTB1 | 1.64E-36 | 0.27226546 | 0.671 | 0.519 | 4.93E-33 | Treg |
| ANXA22 | 2.14E-36 | 0.31351458 | 0.558 | 0.318 | 6.42E-33 | Treg |
| CLIC12 | 4.32E-36 | 0.29624444 | 0.685 | 0.526 | 1.30E-32 | Treg |
| EZR | 3.01E-34 | 0.27220212 | 0.631 | 0.451 | 9.02E-31 | Treg |
| SAT1 | 3.45E-34 | 0.31606177 | 0.567 | 0.4 | 1.04E-30 | Treg |
| UCP2 | 7.78E-34 | 0.3091092 | 0.646 | 0.478 | 2.33E-30 | Treg |
| IL2RA | 1.59E-31 | 0.30432384 | 0.481 | 0.13 | 4.78E-28 | Treg |
| COTL11 | 8.11E-31 | 0.31760189 | 0.618 | 0.475 | 2.43E-27 | Treg |
| ACTG1 | 1.60E-28 | 0.27723786 | 0.651 | 0.523 | 4.80E-25 | Treg |
| HLA-DRA | 6.69E-22 | 0.27560032 | 0.356 | 0.069 | 2.01E-18 | Treg |

**Supplemental Table 1: Differentially expressed genes of 4 major cell types (TN/CM, TEM, Tdiff and Treg).**
